# Supplementary material for: Single-molecule FRET and tracking of transfected biomolecules in living cells
Source: Biophys J. 2025 Sep 19;125(12):3025–33. doi: 10.1016/j.bpj.2025.09.024 (PMC13507326; doi:10.1016/j.bpj.2025.09.024)
Supplement: Supplementary file 1 [file mmc1.pdf]

Biophysical Journal, Volume *125*

Supplemental information

Single-molecule FRET and tracking of transfected biomolecules in living cells

Abhinaya Anandamurugan, Antonia Eidloth, Veronika Frank, Philipp Wortmann, Lukas Schrangl, Chenyang Lan, Gerhard J. Schütz, and Thorsten Hugel

Supplementary Information for: Single-molecule FRET and Tracking of Transfected Biomolecules in Living Cells

Abhinaya Anandamurugan*,†1, Antonia Eidloth*1, Veronika Frank[1], Philipp Wortmann[1], Lukas Schrangl[3], Chenyang Lan[1], Gerhard J. Schütz[3], and Thorsten Hugel†1,2

[1]Institute of Physical Chemistry, University of Freiburg, Albertstr. 21, 79104 Freiburg, Germany
[2]Signalling research centers BIOSS and CIBSS, University of Freiburg, Germany
[3]Institute of Applied Physics, TU Wien, 1060 Vienna, Austria.

September 12, 2025

1. **Suppl. Note 1: FRET efficiency calculations** 2
2. **Suppl. Note 2: Protein production** 5
3. **Suppl. Note 3: Labelling of Biomolecules** 6
4. **Suppl. Note 4: Microinjection** 7
5. **Suppl. Note 5: Tracking Microscopy (HILO)** 9
6. **Suppl. Note 6: Data Analysis** 12
7. **Suppl. Note 7: Example data and S vs E plots** 14

*Equal contribution
†Corresponding authors: th@pc.uni-freiburg.de; abhinaya.anandamurugan@gmail.com

Supplementary Note1: AV and distance calculation

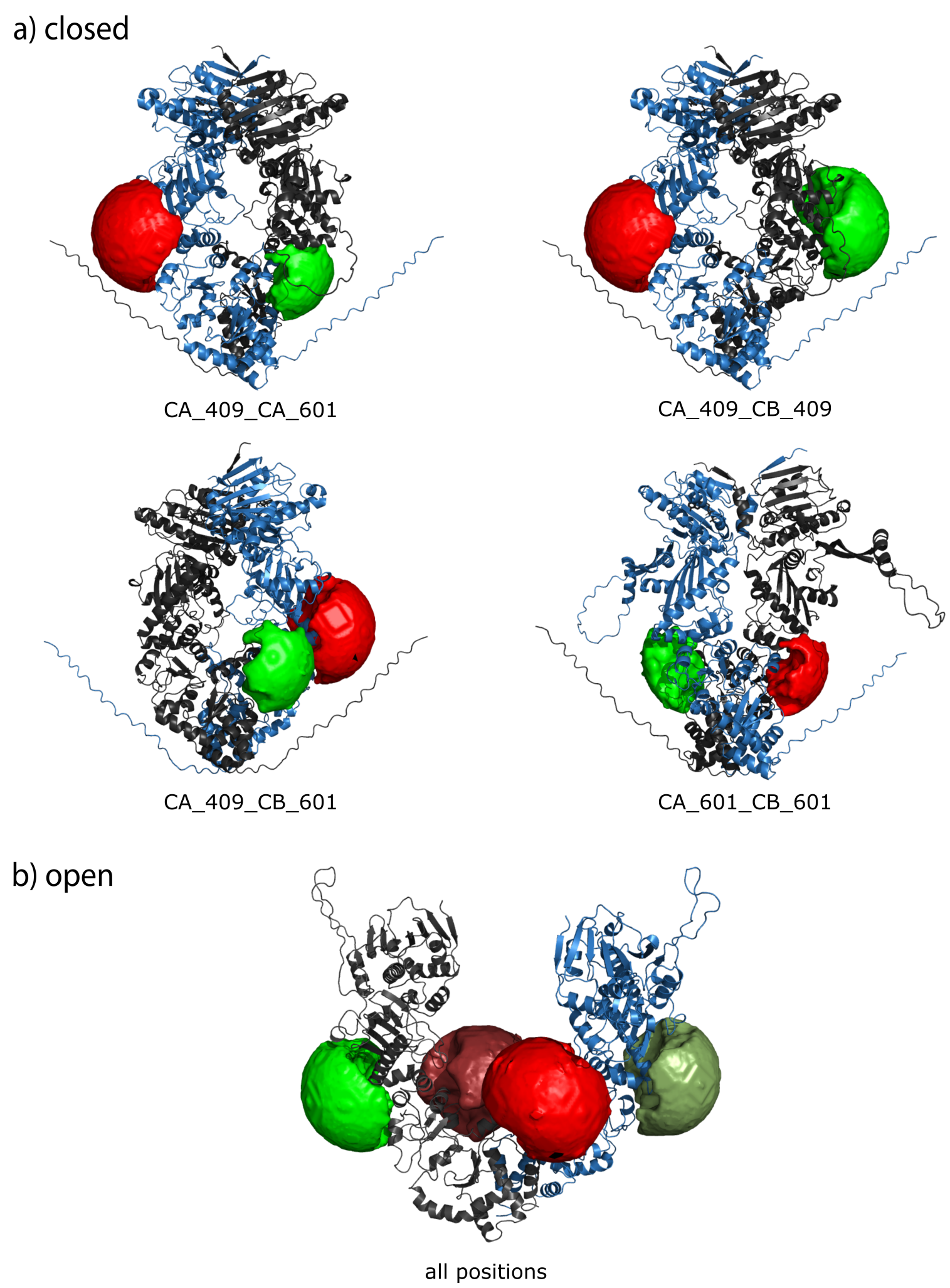

Figure 1: Some possible combinations of labeling positions for the cysteins at positions 409 and 601. Upper four figures based on an Alphafold2 structure for closed yeast Hsp90. Bottom based on the open structure of yeast Hsp90 [1]. Accessible volumes for donor and acceptor are based on FPS analysis [2]. They are depicted in green and red, respectively. CA: Chain A; CB: Chain B.

Accessible dye volume (AV) simulations and distance calculations were performed with the Lumidyne dye pair LD555 and LD655 (Lumidyne Tech). As the original crystal structure of Hsp90 (2cg9) does not fully resolve the C-terminal end, we used the Alphafold2 structure for closed yeast Hsp90. The open struc-

ture of Hsp90 (m-om4) was published before in [1]. Assuming that exactly two dyes are bound to Hsp90, the distances for all possible combinations of dye positions were calculated using the software FPS [2]. The AVs for various combinations are depicted in Suppl.Fig. 1 and the calculated distances and FRET efficiencies are summarized in Suppl. Table 1.

Table 1: Distances for different combinations of labeling positions for the closed Hsp90_409_601 dimer. Calculations were done with dye parameters for LD555 and LD655. Specifications of the two dyes (#1, #2): the dye-position (CA-chain A; CB-chain B) and the kind of dye (A-Acceptor; D-Donor); Rmp-distance between the mean dye positions; $\langle RDA \rangle E$-FRET averaged distance; E-FRET Efficiency.

#1	#2	*Rmp*	$\langle RDA \rangle$	*sigmaDA*	**E**	$\langle RDA \rangle E$
CA_409_A	CA_601_D	63,7	65,3	7	0,435	64,8
CA_409_A	CB_409_D	82,8	84,7	7,1	0,147	83,2
CA_409_A	CB_601_D	33,5	36,3	8,2	0,937	39,5
CA_409_D	CA_601_A	63,7	65,3	7	0,435	64,8
CA_409_D	CB_409_A	82,8	84,7	7,1	0,146	83,2
CA_409_D	CB_601_A	33,5	36,3	8,2	0,937	39,5
CA_601_A	CA_409_D	63,7	65,3	7	0,435	64,8
CA_601_A	CB_409_D	33,5	36,2	8,1	0,938	39,4
CA_601_A	CB_601_D	48,8	49,8	5,9	0,771	50,6
CA_601_D	CA_409_A	63,7	65,3	7	0,435	64,8
CA_601_D	CB_409_A	33,5	36,2	8,1	0,938	39,4
CA_601_D	CB_601_A	48,8	49,8	5,9	0,771	50,6
CB_409_A	CB_601_D	63,8	65,4	7	0,434	64,8
CB_409_A	CA_409_D	82,8	84,8	7,1	0,146	83,2
CB_409_A	CA_601_D	33,5	36,3	8,1	0,938	39,5
CB_409_D	CB_601_A	63,8	65,4	7	0,434	64,8
CB_409_D	CA_409_A	82,8	84,8	7,1	0,146	83,2
CB_409_D	CA_601_A	33,5	36,3	8,1	0,938	39,5
CB_601_A	CB_409_D	63,8	65,4	7	0,434	64,8
CB_601_A	CA_409_D	33,5	36,3	8,2	0,937	39,5
CB_601_A	CA_601_D	48,8	49,9	5,9	0,771	50,6
CB_601_D	CB_409_A	63,8	65,4	7	0,434	64,8
CB_601_D	CA_409_A	33,5	36,3	8,2	0,937	39,5
CB_601_D	CA_601_A	48,8	49,9	5,9	0,771	50,6

Table 2: Distances for different combinations of labeling positions for the open Hsp90_409_601 dimer. Calculations were done with dye parameters for LD555 and LD655. Specifications of the two dyes (#1, #2): the dye-position (CA-chain A; CB-chain B) and the kind of dye (A-Acceptor; D-Donor); Rmp-distance between the mean dye positions; $\langle RDA \rangle E$-FRET averaged distance; E-FRET Efficiency.

#1	#2	Rmp	$\langle RDA \rangle$	$sigmaDA$	**E**	$\langle RDA \rangle E$
CA_409_A	CA_601_D	68,1	70,2	9,9	0,354	68,6
CA_409_A	CB_409_D	111,9	113,4	7,8	0,029	111,5
CA_409_A	CB_601_D	47,5	50,6	8,7	0,742	52
CA_409_D	CA_601_A	68,1	70,2	9,9	0,354	68,5
CA_409_D	CB_409_A	111,9	113,3	7,8	0,029	111,5
CA_409_D	CB_601_A	47,5	50,6	8,7	0,742	52
CA_601_A	CA_409_D	68,1	70,2	9,9	0,354	68,5
CA_601_A	CB_409_D	47,1	50,3	8,8	0,747	51,8
CA_601_A	CB_601_D	33,8	37,4	12,2	0,9	43
CA_601_D	CA_409_A	68,1	70,2	9,9	0,354	68,5
CA_601_D	CB_409_A	47,1	50,3	8,8	0,747	51,8
CA_601_D	CB_601_A	33,8	37,4	12,2	0,9	43
CB_409_A	CB_601_D	67,8	69,8	9,8	0,359	68,3
CB_409_A	CA_409_D	111,9	113,3	7,8	0,029	111,5
CB_409_A	CA_601_D	47,1	50,3	8,8	0,748	51,7
CB_409_D	CB_601_A	67,8	69,9	9,8	0,359	68,3
CB_409_D	CA_409_A	111,9	113,3	7,8	0,029	111,5
CB_409_D	CA_601_A	47,1	50,3	8,8	0,748	51,7
CB_601_A	CB_409_D	67,8	69,9	9,8	0,36	68,2
CB_601_A	CA_409_D	47,5	50,6	8,7	0,742	52
CB_601_A	CA_601_D	33,8	37,4	12,2	0,9	43
CB_601_D	CB_409_A	67,8	69,9	9,8	0,359	68,3
CB_601_D	CA_409_A	47,5	50,6	8,7	0,742	52
CB_601_D	CA_601_A	33,8	37,4	12,2	0,9	43

Supplementary Note 2: Protein production

Hsp90z_Avi_409C_601C: Cysteine residues were introduced into the sequence of yeast Hsp90 via site-directed mutagenesis at positions 409 and 601 (Quick Change Lighting kit, Agilent). The cysteine modified Hsp90 was expressed with a pET28 vector. Additionally this protein has an Avi-tag, which leads to a biotinylation in the bacterial cell with the help of biotin ligase enzymes co-expressed in a pBirAcm (Avidity Nanomedicine, La Jolla, CA) plasmid. The cells were grown in LB media supplemented with 0.5% glucose and Kanamycin (100µg/ml) at 37 °C. At a OD of 0.7, the expression was induced by adding isopropyl β-D-1-thiogalactopyranoside (IPTG; Sigma; final concentration 1 M). Furthermore, biotin (prepared in 10 mM Bicine Buffer pH 8.3 (adjusted with 1 M KOH, filter sterilized)) was added to the medium with a final concentration of 50 μM. The cells were then harvested 4 hours after induction and pelleted by centrifugation at 6000 rpm. Cell pellets are stored at $-20°$C until protein purification. For cell lysis pellet was resuspended in NiNTA A buffer with 20 mM imidazole. Protease inhibitor 50x is added prior to cell disruption in a cell disruptor (I & L Biosystems GmbH). $MgCl_2$ (final concentration 2 mM) and 6 µl of Benzonase (EMD Millipore Corp, USA) are added to the cell lysate. It is then centrifuged for 1 h at 50,000g. The supernatant is then passed through a 0.45 filter (Merck Millipore millex), the filtrate is then collected and fed to the HisTrap. The filtrate collected is then fed to the sample pump for HisTrap1. The NiNTA HisTrap column is pre-washed and equilibrated with HisA1 buffer. The sample is loaded onto the column and then allowed to run through the HisTrap1. The bound fraction is collected. The outlet collected from HisTrap1 is fed again into the HisTrap2. The bound fraction is again collected and diluted so the total salt concentration is less than 30mM. This sample is then fed into HiTrapQ. The eluted fraction is then collected and then concentrated to less than 3 g/l and injected into the Size exclusion column 26/600. The final purified samples are collected, concentrated and frozen in liquid nitrogen for later use.

Supplementary Note 3: Labeling of biomolecules

The protein is labelled based on cysteine maleimide chemistry. The protein is reduced with 1 mM Tris (2-carboxyethyl) phosphine (TCEP) pH 7.0 for 30 minutes at room temperature. Then the protein is equilibrated with ice cold phosphate saline buffer pH 6.7 by washing in a centrifugal 50kDa cut-off spin filter (3 times) which removes excess TCEP. Then 1-2 fold (mol) of dye (LD555 and LD655) is added. First the acceptor dye (LD655) is added and incubated for 2 minutes, then the donor dye (LD555) is added. The mix is incubated at room temperature for 2 h. The labelled protein is then purified with PD MiniTrap G25 columns (Cytiva). The column is washed thrice with Tyrode's buffer. Then the protein is added and the column centrifuged for 2 min, at 1000 g. The flow through was collected and used for the experiments.

To create heterodimers with one doubly labeled monomer and one unlabeled monomer, a monomer exchange was performed. The labeled protein is mixed with an unlabeled variant with a C-terminal zipper (Hsp90z_wt). A ratio of 1:20 (labeled:unlabeled) is used and final concentrations of 2 μM and 80 μM in Tyrode's buffer are used. The proteins are incubated at 43°C for 36 min with shaking at 300rpm in a thermocycler (Eppendorf). The heating destabilizes the C-terminal zipper and enables exchange of the monomers. Possible aggregates are removed subsequently by centrifugation at 14,000 g and 4 °C for 30 min.

Supplementary Note 4: Microinjection

Cell viability was tested by using pHrodo Red and Green AM intracellular pH indicator dyes (ThermoFisher Scientific). The cells used for injection were first calibrated for the dye response with different pH buffer conditions using the Intracellular pH calibration Buffer kit (ThermoFisher Scientific). Imaging of the cells was done on an Olympus microscope fitted with a HE90 dichroic cube. Images of the cell area of the different dishes were taken before dye addition. After addition of dye, washing and incubation with pH calibration buffers for the respective cell dishes an image of the same area was recorded. For cell viability test new cell dishes were taken and areas for injection were imaged in OptiMEM before injection. The following injection of cells were made with H1 buffer, then the cells were washed with preheated PBS (twice) and imaged in optiMEM. Images of the cell area of the different dishes was taken before dye addition. After addition of dye, washing and incubation with OptiMEM the respective areas of dishes were imaged. Uninjected area cells were also imaged as a control. For intensity measurements, mean of 10 cells were taken for each of the three time points (1st hour, 2nd hour, 3rd hour) for the 4 different pH measurements (pH 4.5, pH 5.5, pH 6.5, pH 7.5). For before and after measurements care was taken to measure the same cells and same region. All measurements were background corrected.

**Parameter**	**Value**
Speed (Coarse-mode) (µm/sec)	4000
Step injection speed (µm/sec)	50
Step injection distance (µm/sec)	20
Position speed (µm/sec)	1500
Injection axial	ON
Piezoxp. Speed (µm/sec)	300
Piezoxp. Distance (µm)	20
Piezoxp. axial	OFF
Angle of injection	45°

Table 3: Injector Parameters

Fixed cell experiments: The purified DNA/protein sample of 1nM is prepared in a vial with degassed buffer which is double filtered. Injection needle is filled with 5 µl of 1nM sample and fitted to the injector nozzle. Before injection, the cell area to be injected is imaged in the DIC, Green (555nm), Red(630nm) emission channels of the fluorescence microscope to check for any leakage or fluorescence abnormalities. The grid number on the ibidi dish is noted. The cells are then washed twice with DPBS 1ml (pre-warmed at 37°C) and then 1 ml of Opti-MEM is added, then cells within the marked grid area are injected. After injections, cell area within the grid is imaged again with DIC, green and red channels to confirm no leakage. Then the Opti-MEM is removed, cells washed with DBPS twice followed by addition of 500 µl of 4 percent Paraformaldehyde

(PFA). The dish is incubated for 20 mins in dark at room temperature. Then the PFA is washed twice with in DPBS and 2ml of Opti-MEM was added. The cells were then ready to be imaged.

Supplementary Note 5: HILO microscopy

For all *in cellula* measurements a home-built HILO microscope as depicted in Suppl.Fig. 2 was used. A theoretical depth of view calculation was done to estimate the minimum frame requirement for a molecule to be visible with a 70 ms exposure time and a certain diffusion constant. For a 100x Nikon objective with a 1.49 NA, the theoretical depth of field is 190 nm. The calculated depth of field d_{tot} as per the below parameters was found to be:

$$d_{\mathrm{tot}} = \frac{\lambda n}{\mathrm{NA}^2} + \frac{n \cdot e}{M \cdot \mathrm{NA}} \tag{1}$$

Substituting given values:

$$d_{\mathrm{tot}} = \frac{553\,\mathrm{nm} \cdot 1.515}{1.49^2} + \frac{1.515 \cdot 14\,\mu\mathrm{m}}{200 \cdot 1.49} = 449\,\mathrm{nm} \tag{2}$$

Here, the refractive index $n = 1.515$; wavelength $\lambda = 553\,\mathrm{nm}$; the variable $e = 14\,\mu\mathrm{m}$ is the smallest distance that can be resolved by a detector placed in the image plane of the microscope objective, whose lateral magnification is $M = 200$. In the following we estimate the displacement of a single molecule (in z-direction) that can still be tracked, x_{tr}, assuming the molecule is first detected in the center of the focus. We start from the depth of field of the microscope as:

$$x_{\mathrm{tr}} \approx \frac{1}{2} d_{\mathrm{tot}} \tag{3}$$

Substituting (2) in (3), $x_{\mathrm{tr}} \approx 224\,\mathrm{nm}$ is the maximum possible mean squared displacement, where the molecule can be tracked, i.e., beyond this distance the particle might leave the focus and cannot be tracked. The mean squared displacement in one dimension, x^2, of a molecule is related to the diffusion coefficient D for a time t as:

$$t \approx \frac{x^2}{2D} \tag{4}$$

$$x \approx \sqrt{2Dt} \tag{5}$$

We can now calculate the mean squared displacement for a given diffusion constant and time. E.g. for $D = 0.05\mu\mathrm{m}^2/\mathrm{s}$ and t=10s we would obtain $x = 33$ nm. Therefore, most molecules would not leave the focus for such an experiment. **Correction factors** were estimated for esDNA and Hsp90_409C_601C. Both were labelled with LD655 and LD555 and both were biotinylated. The esDNA and Hsp90 were separately immobilized on PEG surfaces coated with Biotin-NHS PEG. Then the molecules were attached to the surface via biotin-Neutravidin binding and measured in H1 buffer pH 7.5 with Tris (5 mM Tris, 20 mM $MgCl_2$, 5 mM NaCl, pH 7.5) at 10pM for esDNA. The measurements were carried out with ALEX. The movies were analyzed using homewritten procedures based on IgorPro (wavemetrics), which is detailed in [3]. The obtained correction factors are given in Supplementary Table 6.

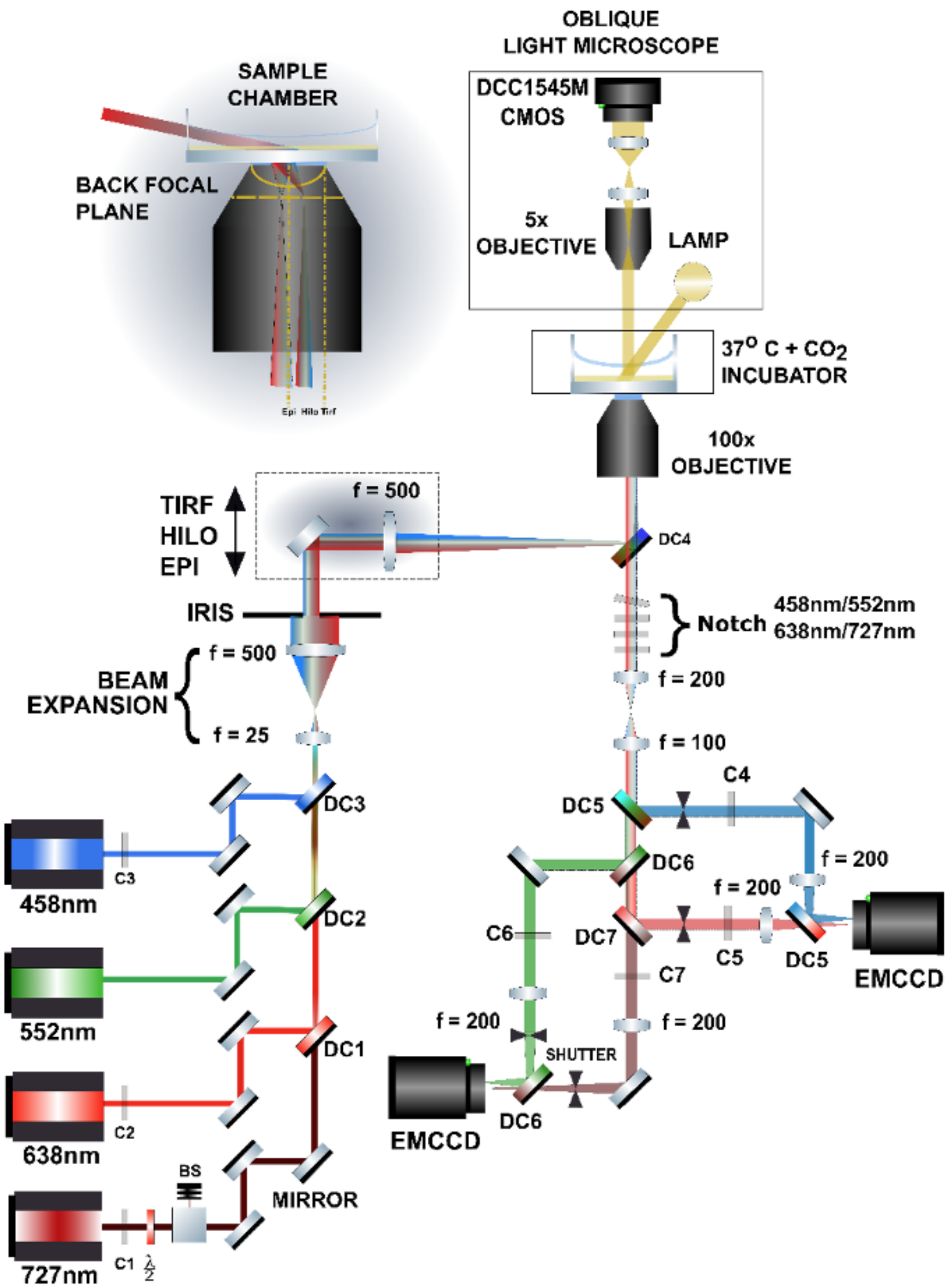
OBLIQUE
LIGHT MICROSCOPE
DCC1545M
CMOS
5x
OBJECTIVE
LAMP
SAMPLE
CHAMBER
BACK FOCAL
PLANE
37° C + CO2
INCUBATOR
100x
OBJECTIVE
TIRF
HILO
EPI
f = 500
DC4
IRIS
Notch
458nm/552nm
638nm/727nm
f = 500
BEAM
EXPANSION
f = 25
f = 200
f = 100
C4
DC5
DC3
458nm
C3
DC6
f = 200
f = 200
DC2
C6
DC7
C5
DC5
EMCCD
552nm
C7
f = 200
f = 200
DC1
SHUTTER
638nm
C2
EMCCD
DC6
BS
MIRROR
727nm
C1

Figure 2: Schematic representation of the 4-color HILO microscope equipped with an oblique light microscope for viewing cell injection area. All the optical and live-cell components are mounted on a vibration-damping optical table (RS2000, Newport). The following four lasers are used in our custom-built setup: 458nm (Obis 458LX, Coherent), 638nm (Obis 637LX, Coherent), 552nm (Obis 552LS, Coherent), 727nm (Obis 730LX, Coherent). The lasers are cleaned up using clearance filters and then overlaid using specific dichroic mirrors DC1, DC2 and DC3. An iris is positioned to select homogeneous illumination for all overlaid lasers. Subsequently the beam is expanded 20x by a Keplerian telescope and further a 2-inch iris cuts out the central illumination region. Further this beam is focused on to the back focal plane of the objective via a multiband Quad Line beam splitter DC4 using a 2-inch cylindrical lens mounted on a translation stage. This translation stage helps to shift the light path away from the optical axis (epi) to HILO and TIRF regions. The objective used is (CFI Apo TIRF 100x, Nikon) with NA=1.49. A set of notch filters are setup after the Quad Line beam splitter to block the excitation beam. The emission fluorescence is then directed to the detection box where the beam passes through a Keplerian lens with an adjustable optical slit (Owis GmbH). This helps to remove any off-axis beams and increases the magnification by a factor 2. Laser wavelengths are separated by dichroic mirrors DC5, DC6 and DC7. Corresponding emission filters C4, C5, C6, and C7 are placed in the emission pathway to cut off any residual excitation light. Additionally, a SH05 Ø1/2" Beam Shutter (Thorlabs) is placed after each emission filter to prevent bleed-through. The wavelengths 485nm and 637nm are separated from 552nm and 738nm and then focused separately on two EMCCD cameras (Xion Ultra 897 EX2 dua AR coated with fringe suppression, Andor Technology Ltd.) to facilitate multiple FRET-pair imaging. Finally, each beam is focused on to the full chip of the EMCCD detectors with aspheric lenses (Qioptiq) with 50.8 mm diameter. Sample chambers designed to integrate temperature, CO2 and humidity control (Okolab, Naples, Italy) are mounted on the microscope stage. For live-cell smFRET imaging, cells were grown in ibidi μ-dish 500 grid glass-bottom plates (ibidi GmbH, Munich). Filters: C1 - FL457.9/10 (ThorLabs), C2 - ZET635/10, C3 - 720/24 BrightLine HC, C4 - 525/50 BrightLine HC, C5 - 675/50 ET, C6 - 593/46 BrightLine HC, C7 - 810/90 ET Bandpass (rest from AHF Analysentechnik). Dichroics: DC1 - BS zt 670 rdc-xxrxt, DC2 - BS zt 561 RDC xrxt, DC3 - BS zt 473 RDC, DC4 - zt488/543/635/730rpc flat, DC5 - T 556 LPXR, DC6 - HC BS 649, DC7 - HC BS 740 (all from AHF Analysentechnik). Notch filters: 458 nm - ZET457NF,638 nm - ZET635NF, 727nm - ZET730NF (all from AHF Analysentechnik).

Supplementary Note 6: Data Analysis

The data analysis is done with a python based code called fret-analysis, previously described in [4] and available on GitHub. Analysis starts by running the Jupyter Notebook titled "01. Tracking". First, the microscope movies (TIFF file stacks) must be provided along with calibration files for the laser profile and channel alignment. Channel alignment is done prior to tracking. For this, threshold parameters for feature radius, mass and signal intensity need to be set so that fiducial markers (e.g. fluorescent beads) in the registration calibration file are recognized. The same procedure is repeated for the actual data files. Parameters should be chosen to locate single-molecule signals while minimizing the recognition of unspecific background fluorescence. The parameters used in this study are listed in table 4. For tracking the Crocker-Grier algorithm was chosen. Several tracking parameters can be adjusted (see table 5). The tracking results are saved into six separate files. Next, the notebook "02. Analysis AE" is executed. This notebook is based on the notebook "02. Analysis" provided in [4]. However, the built-in data correction is skipped (set $\alpha = 0, \delta = 0, \beta = 1, \gamma = 1$), because instead we employ our home written Igor Pro Software for correction. The updated data is again saved into six files. A manual trace selection is then carried out using the GUI tool "inspector_gui", which can be launched from the command line (python -m smfret_analysis.inspector_gui). This tool allows for visual inspection of each recognized and tracked particle. An overview of the following information is shown for each particle: its position in the three separate movies (donor, FRET and acceptor); intensity traces over time for each channel; and the SE plot. Based on this information, users can manually accept or reject traces for further analysis. Selections are saved by clicking the appropriate button in the interface. In the third step, the selected traces are exported as text files. This is done by running the adjusted notebook "03. Plots" up to the plot_summary function with the export="True" argument. These .txt files are subsequently used for further analysis in Igor Pro. To import the traces into Igor Pro, the function "Importer_ascii()" is used. To access the data the "SetNotes()" function must be called. The traces can then be viewed via the "show saved traces" panel. Each trace is then manually segmented into three regions: 1. Potential FRET – both dyes are present. 2. Single dye – one dye has bleached. 3. Background – both dyes have bleached. From this segmentation, SE 2D histograms and the corresponding 1D histograms can be generated. Correction can be done interactively. In this study, the correction factors were determined with in vitro measurements of esDNA, see Table 6.

Table 4: Localization and Tracking Parameters.

Parameter	Donor	Acceptor
**Registration options**		
Algorithm	Crocker-Grier	Crocker-Grier
Radius	2	2
Signal Threshold	1000	500
Mass Threshold	1000	1000
**Localization options**		
Algorithm	Crocker-Grier	Crocker-Grier
Radius	2	2
Signal Threshold	1000	500
Mass Threshold	20000	13000

Table 5: Linking of Particles Parameters.

Parameter	Value
Feature radius (feat_radius)	2
Link memory (link_mem)	20
Minimum length (min_length)	20
Background frame (bg_frame)	3
Background estimator (bg_estimator)	mean
Lag time	infinity

Table 6: Analysis Correction Factors.

Correction factors	HILO	Confocal
Leakage α	0.17	0.23
Direct Excitation δ	0.12	0.05
Excitation Efficiency β	1	0.7
Detection Efficiency γ	0.8	1

Supplementary Note 7: Example data and S vs E plots

In Suppl. Fig. 3 we show full distributions (S vs E plots and projections) for the DNA samples in vitor, in fixed cells and in live cells. Suppl. Fig. 4 shows additional example traces for DNA in living cells and suppl. Fig. 5 for Hsp90 in living cells.

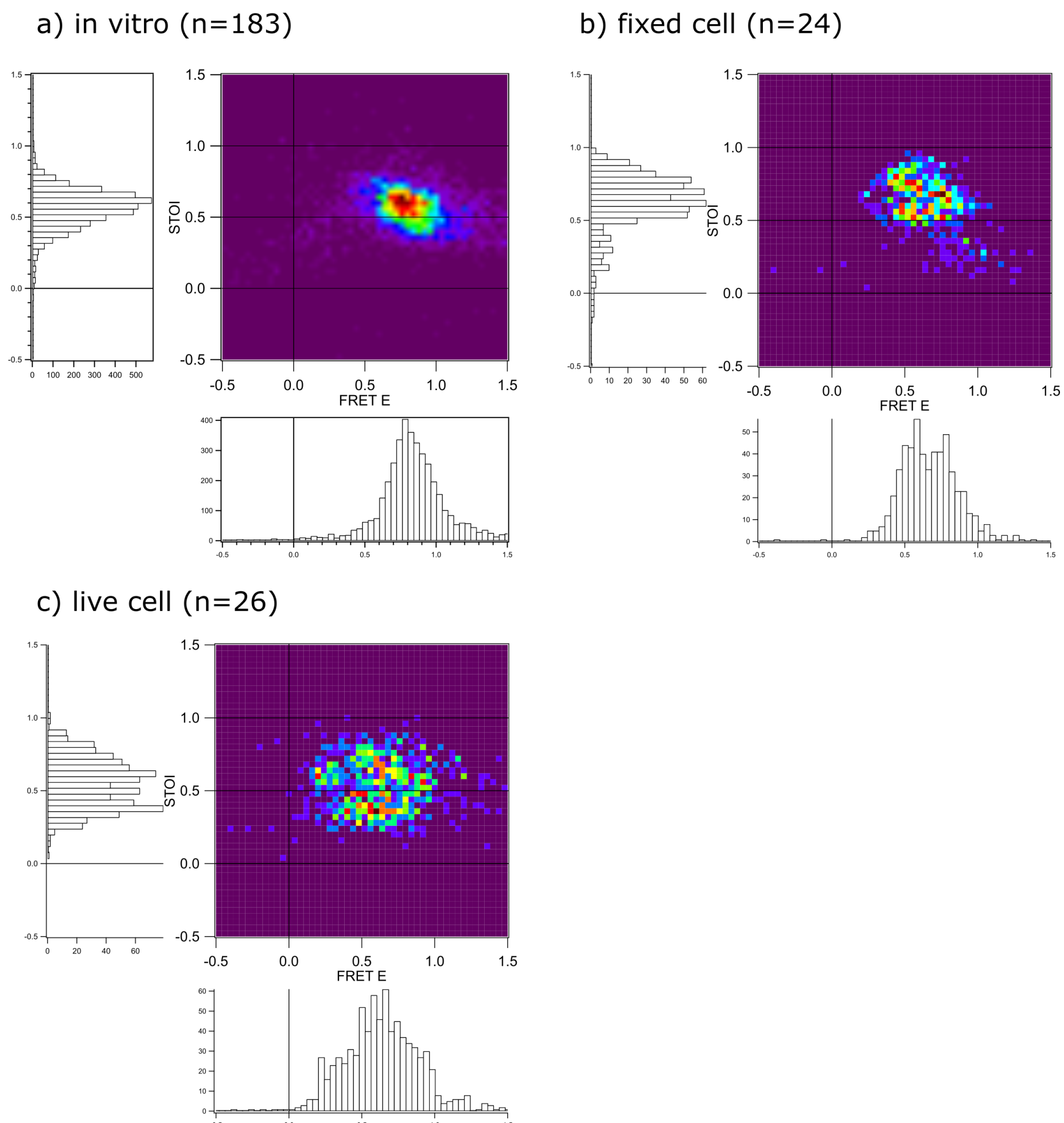

Figure 3: Combined distribution of esDNA smFRET data. a) In vitro data from a total of 183 traces obtained from 14 movies; b) Fixed cell data from a total of 24 traces obtained from 9 cells; c) Live cell data from a total of 51 cells. 1D histograms of stoichiometry (each left) and FRET efficiency (each bottom) and 2D histograms of stoichiometry vs FRET efficiency (each middle panel). Correction factors were applied.

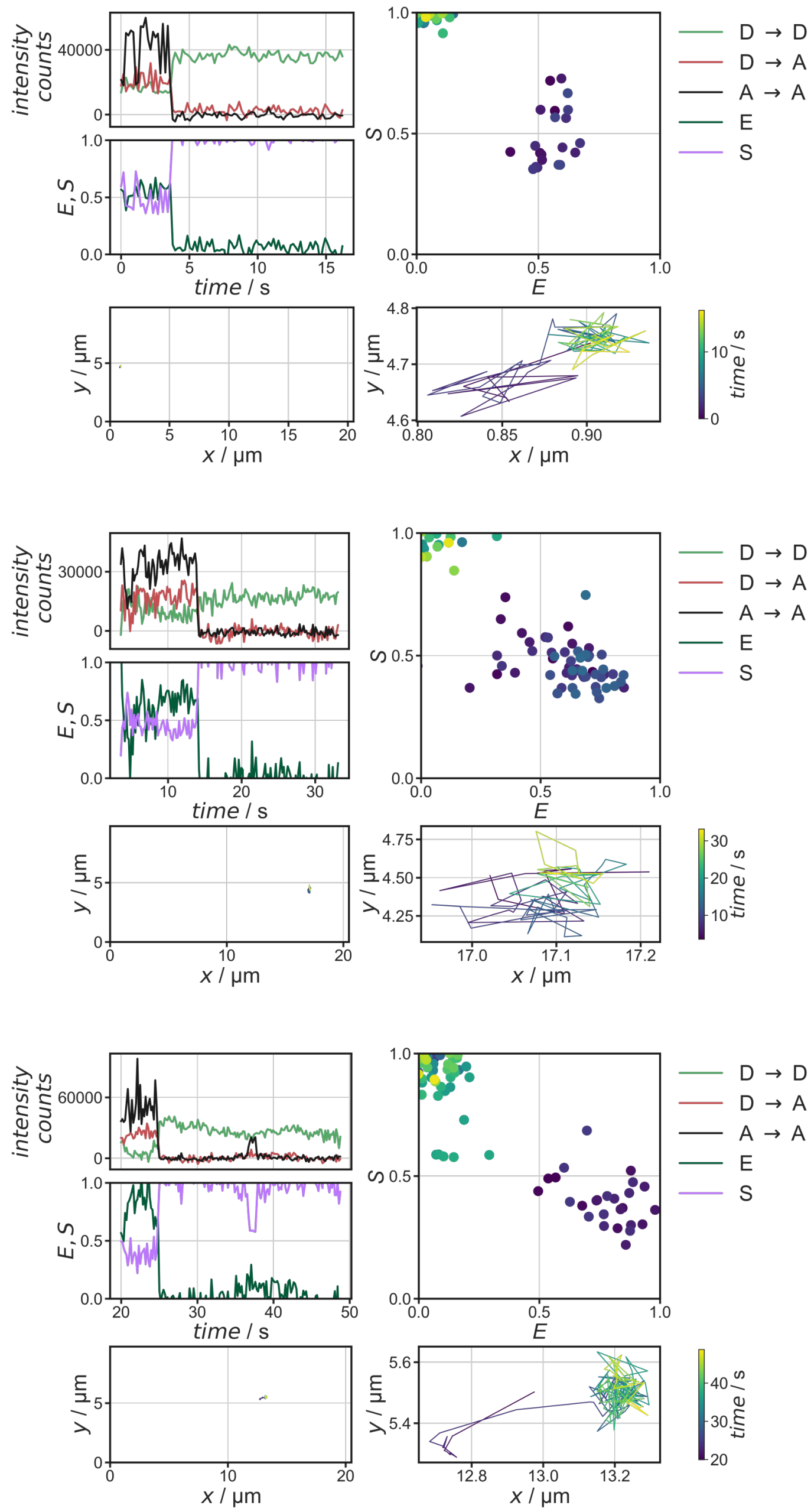

Figure 4: Example traces of live-cell smFRET measurements with esDNA. Intensity in donor, FRET and acceptor as well as FRET efficiency and stoichiometry over time. Corresponding E vs S plot and tracks. Data shown is raw data, i.e. no correction factors were applied. The top figure likely shows blinking of a second acceptor during the first four seconds, which demonstrates how important the ALEX is.

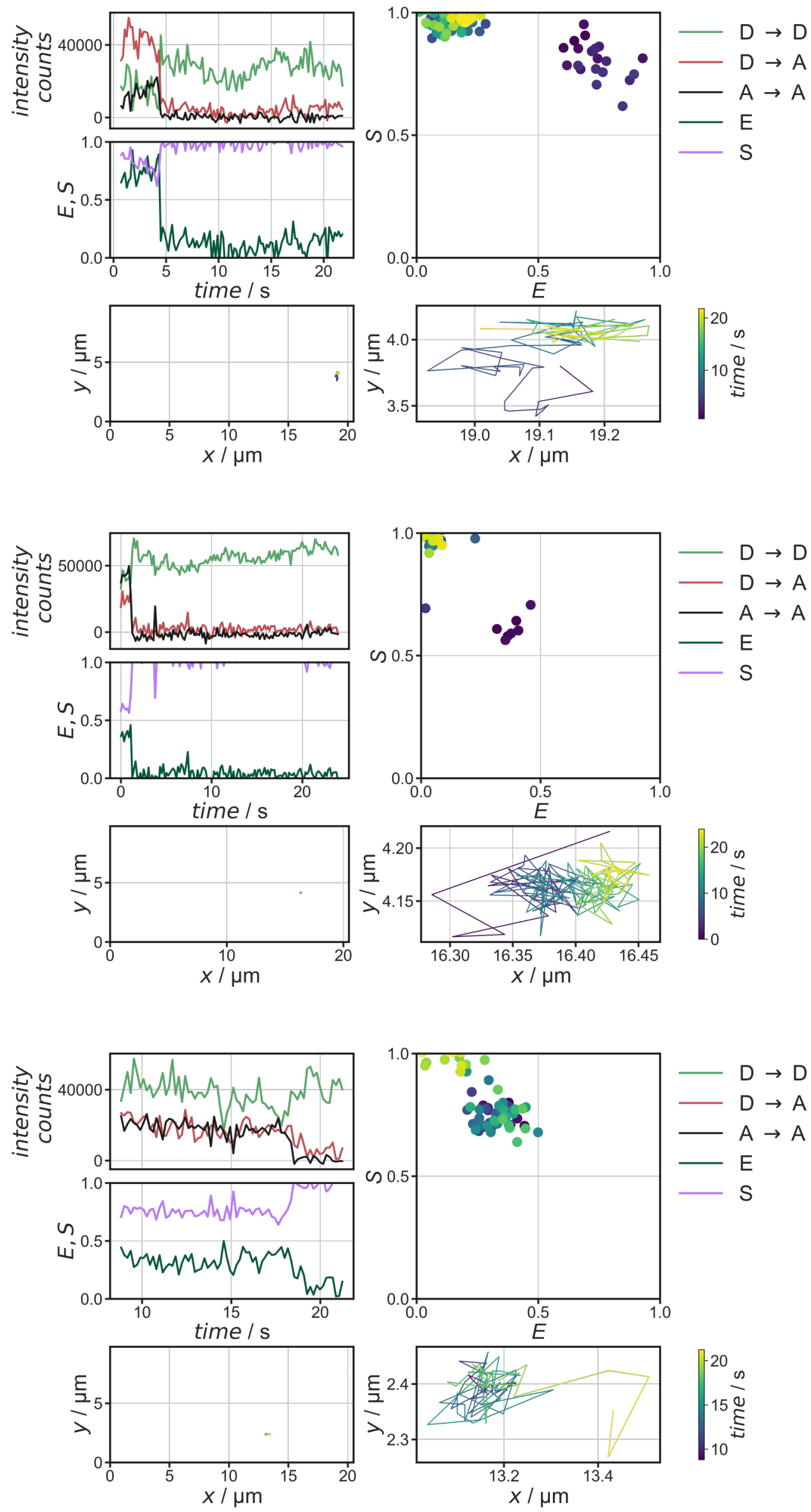

Figure 5: Example traces of live-cell smFRET measurements with Hsp90_409_601. Intensity in donor, FRET and acceptor as well as FRET efficiency and stoichiometry over time. Corresponding E vs S plot and tracks. Data shown is raw data, i.e. no correction factors were applied. This might in part explain the higher stoichiometry as expected (S around 0.5 is expected). Especially the beta factor (which affects S significantly) is difficult to determine.
