# Supplementary material for: Single-molecule FRET and tracking of transfected biomolecules in living cells
Source: Biophys J. 2025 Sep 19;125(12):3025–33. doi: 10.1016/j.bpj.2025.09.024 (PMC13507326; doi:10.1016/j.bpj.2025.09.024)
Supplement: Supplementary file 2 [file mmc2.pdf]

Biophysical Journal
Article

Biophysical Society

Single-molecule FRET and tracking of transfected biomolecules in living cells

Abhinaya Anandamurugan,[1,*] Antonia Eidloth,[1] Veronika Frank,[1] Philipp Wortmann,[1] Lukas Schrangl,[3] Chenyang Lan,[1] Gerhard J. Schütz,[3] and Thorsten Hugel[1,2,*]
[1]Institute of Physical Chemistry, University of Freiburg, Freiburg, Germany; [2]Signalling Research Centers BIOSS and CIBSS, University of Freiburg, Freiburg, Germany; and [3]Institute of Applied Physics, TU Wien, Vienna, Austria

ABSTRACT Proteins and DNA in cells exhibit different conformational states, which are influenced by dynamic interactions with other biomolecules. All these interactions are affected by the molecules' localization within the cell, i.e., their compartmentalization. Such, in cellula, compartment-specific dynamics is difficult to measure, because of limitations in instrumentation, autofluorescence of cells, and the necessity to track diffusing molecules. Here, we present a bottom-up engineering approach, which allows us to track transfected proteins in cellula and to analyze time-resolved single-molecule FRET efficiencies. This has been achieved by alternating laser excitation-based dual-channel (donor and FRET, acceptor) tracking with a HILO microscope. We validate our strategy by characterizing long-term static FRET traces of customized DNA with known dye positions. We utilize two different transfection strategies, namely microinjection (physical) and a transfection mediated by the toxin Streptolysin-O (biological). By comparing in vitro and in cellula measurements we show that the cellular environment in this case changes the FRET efficiency by about 25%. In addition, we evaluate single-molecule FRET traces for the heat shock protein Hsp90 in cellula. The obtained FRET efficiency distribution is largely consistent with known Hsp90 structures and in vitro distributions, but also shows some clear differences. Altogether, we show that FRET-TTB opens the path to study protein state changes of transfected biomolecules in living cells, including their time-resolved cellular localization.

SIGNIFICANCE Proteins and DNA are constantly changing their shape, influenced by the environment and interactions with other molecules. Studying these changes at the single-molecule level in living cells is challenging due to the motility of the molecules and the complexity of the cytoplasmic environment. Here, we demonstrate a technique called FRET-TTB that enables us to track individual proteins inside living cells over time using single-molecule fluorescence microscopy. We validate the approach with custom DNA and apply it to study the heat shock protein Hsp90. Our results show that this method can reveal changes in protein conformation and location within living cells.

INTRODUCTION

Investigation of protein structure and dynamics in vitro has largely enhanced our understanding of proteins and their interactions. Determining how protein structure, dynamics, and interactions are affected in a crowded nonequilibrium environment, such as the cytosol of living cells, in a spatially resolved way poses the next challenge. In vitro, long-term smFRET measurements have been most reliable in detecting dynamic distance changes of less than 10 nm (1). Consequently, a promising direction is to extend smFRET measurements to live-cell applications. For some membrane proteins, this has already been achieved (2), but not for the recruitment and release of proteins to and from membranes (3) or for cytosolic proteins such as Hsp90. The central problem to achieve this in mammalian cells is to track single molecules showing conformational dynamics and to disentangle the effect of the microenvironment from protein conformational state changes. Two main types of smFRET methods have been employed to study conformational changes: lifetime photon burst measurements with confocal microscopy, and fluorescence intensity measurements with total internal reflection microscopy or highly inclined laminated optical sheet (HILO) microscopy.

Submitted May 31, 2025, and accepted for publication September 16, 2025.
*Correspondence: abhinaya.anandamurugan@gmail.com or th@pc.uni-freiburg.de
Philipp Wortmann's present address is AstraZeneca, Munich, Germany
Chenyang Lan's present address is PicoQuant, Berlin, Germany
Abhinaya Anandamurugan and Antonia Eidloth contributed equally to this work.
Editor: Joachim Raedler.
https://doi.org/10.1016/j.bpj.2025.09.024
© 2025 The Author(s). Published by Elsevier Inc. on behalf of Biophysical Society.
This is an open access article under the CC BY license (http://creativecommons.org/licenses/by/4.0/).

Lifetime measurements provide rich information on conformations of free or immobile proteins (4), but they are not suitable for long-term measurements that follow one single molecule over time. Intensity-based two-color smFRET is generally suitable for long-term measurements on a single molecule, but only if the molecule is immobilized (5). Therefore, it can also not be directly applied for mobile molecules in living cells. Fluorescence correlation spectroscopy on the other hand has been widely used to study diffusion in biological systems (6), but its application to study conformational changes is still in its infancy. The challenge is to localize both single fluorophores, the donor and the acceptor, track them, and determine the FRET efficiency and stoichiometry over time. Here, we present a method that meets this challenge in cellula by establishing long-term smFRET measurements of the cytosolic protein Hsp90 using HILO microscopy (7), dual-channel tracking (donor and FRET, acceptor; see materials and methods for details) (8), and cell transfection. The two channels are the result of alternating laser excitation (ALEX) (9,10), which is useful to recognize the fluorophores' photophysics. The workflow for this method is shown in Fig. 1. Here and in the following, the donor signal is the signal intensity in the donor channel upon donor excitation ($I_{\mathrm{Dem|Dex}}$), FRET the intensity in the acceptor channel upon donor excitation ($I_{\mathrm{Aem|Dex}}$), and the acceptor signal the intensity in the acceptor channel upon acceptor excitation ($I_{\mathrm{Aem|Aex}}$). Key requirements for tracking and obtaining long-term intensity traces of single molecules are photophysically stable FRET dye-pairs and low intracellular concentrations of the labeled molecules. This enables clear separation of signals, detectable for a long period of time. Our strategy to achieve this is in vitro labeling of purified proteins with synthetic high-performance dyes and the subsequent transfection of cells with the prelabeled molecules. This allows for a high degree of control over the final amount of labeled molecules inside the cell and therefore helps to maintain single-molecule concentrations (11–13). Additionally, and in contrast to endogenously expressed fluorescent proteins, the cell's native cellular expression is preserved. However, when proteins are labeled in vitro, they have to be delivered to the cells' inside afterward. We tested and compared two mechanistically different transfection strategies: a biological method using the pore-forming toxin Streptolysin-O (SLO) (14–17) and a physical method, namely microinjection (4,18,19). Both have the capability to introduce biomolecules with a high molecular weight, such as the yeast Hsp90 homodimer with a molecular weight of about 164 kDa per dimer. We first benchmark the power of our FRET-TTB (Förster resonance energy transfer-tracking of transfected biomolecules) approach and the transfection strategies by quantifying single-molecule FRET of customized DNA oligos with known dye distances. We then show that this approach with SLO transfection also works for the molecular chaperone and heat shock protein Hsp90. Due to its role as a molecular chaperone, the resident cytosolic Hsp90 presents a hub for protein interactions and is involved in multiple proteomic pathways (20,21). Notably, in vitro single-molecule studies showed that the Hsp90 dimer occupies two major, dynamically exchanging conformational states, namely an N-terminal open and a closed state (22,23). However, the relation between the dynamic conformational state changes and Hsp90's biological functions is still elusive (24). Live-cell smFRET measurements are ideal for addressing this, enabling to watch Hsp90 at work in its native environment. Employing our approach we were already able to follow single molecules inside living HeLa cells for up to 89 s and quantify smFRET for more than 20 s. A comparison of the quantified live-cell FRET efficiencies with in vitro results and theoretical FRET efficiencies shows good agreement and therefore proves the method successful. This opens up new ways to study how proteins function in their natural environment.

MATERIALS AND METHODS

DNA and protein preparation

To understand the quality of the obtained smFRET traces, we first analyzed standard DNA, previously used in in vitro smFRET experiments and benchmarked in (25). To prevent intracellular degradation, we used modified, end-sealed DNA, labeled with Lumidyne dyes LD555 and LD655 (*ATD Bio*). Here, the ends of the DNA oligo are ligated with the use of copper-based click-chemistry. Yeast Hsp90 was designed with a C-terminal zipper motif (coiled-coil motif of DmKHC, *D. melanogaster*) for constitutive dimerization and the two point mutations E409C and S601C were introduced for site-specific labeling. This construct is then named *Hsp90z_E409C_S601C*. The positions for labeling were chosen to ensure a stable FRET signal, remaining mostly unaffected by the pronounced conformational rearrangements between the two monomers (26). Theoretical FRET efficiency values are obtained from accessible volume simulations and distance calculations, done with the FRET positioning and screening software (27). The calculations are described in Note S1 and the results are summarized in Tables S1 and S2. Hsp90 was expressed and purified as described in Note S2 and then labeled via cysteine-maleimide chemistry with Lumidyne dyes (LD555 and LD655) as described in Note S3. It is to be noted that specific dual labeling of Hsp90 is achieved via monomer exchange with a 20-fold excess of unlabeled wild-type Hsp90.

Transfection of biomolecules

Microinjection or a transfection with the toxin SLO were used to introduce labeled molecules in living HeLa cells. For microinjection, cells were grown to confluency at 37°C in cell culture imaging dishes (μ-Dish 35 mm, high Grid-500 Glass Bottom, Ibidi, Gräfelfing, Germany). The injection was monitored with a fluorescence microscope (Axio Observer 3, Carl Zeiss Microscopy, Oberkochen, Germany) equipped with a DIC module, a Colibri 7 LED light source, beam splitters, and a 20× Zeiss air objective. The injection system Femtojet 4i (Eppendorf, Hamburg, Germany) was used in combination with the micromanipulator InjectMan 4 (Eppendorf). Before injection, the cells were washed twice with preheated DPBS and then Opti-MEM was added. The samples were diluted in H1 buffer (40 mM HEPES, 150 mM KCl, 10 mM $MgCl_2$ [pH 7.4]). The injection needles Femtotip II (Eppendorf) were filled with the protein sample using microloader pipette tips. Injections were performed with the following settings: injection pressure = 300 hPa, time = 0.5 s, and compensation pressure = 30 hPa. Postinjection, Opti-MEM was removed and the cells washed twice with DBPS. For fixation, 500 μL of 4% paraformaldehyde was added. Then the dish was incubated for 20 min at room temperature.

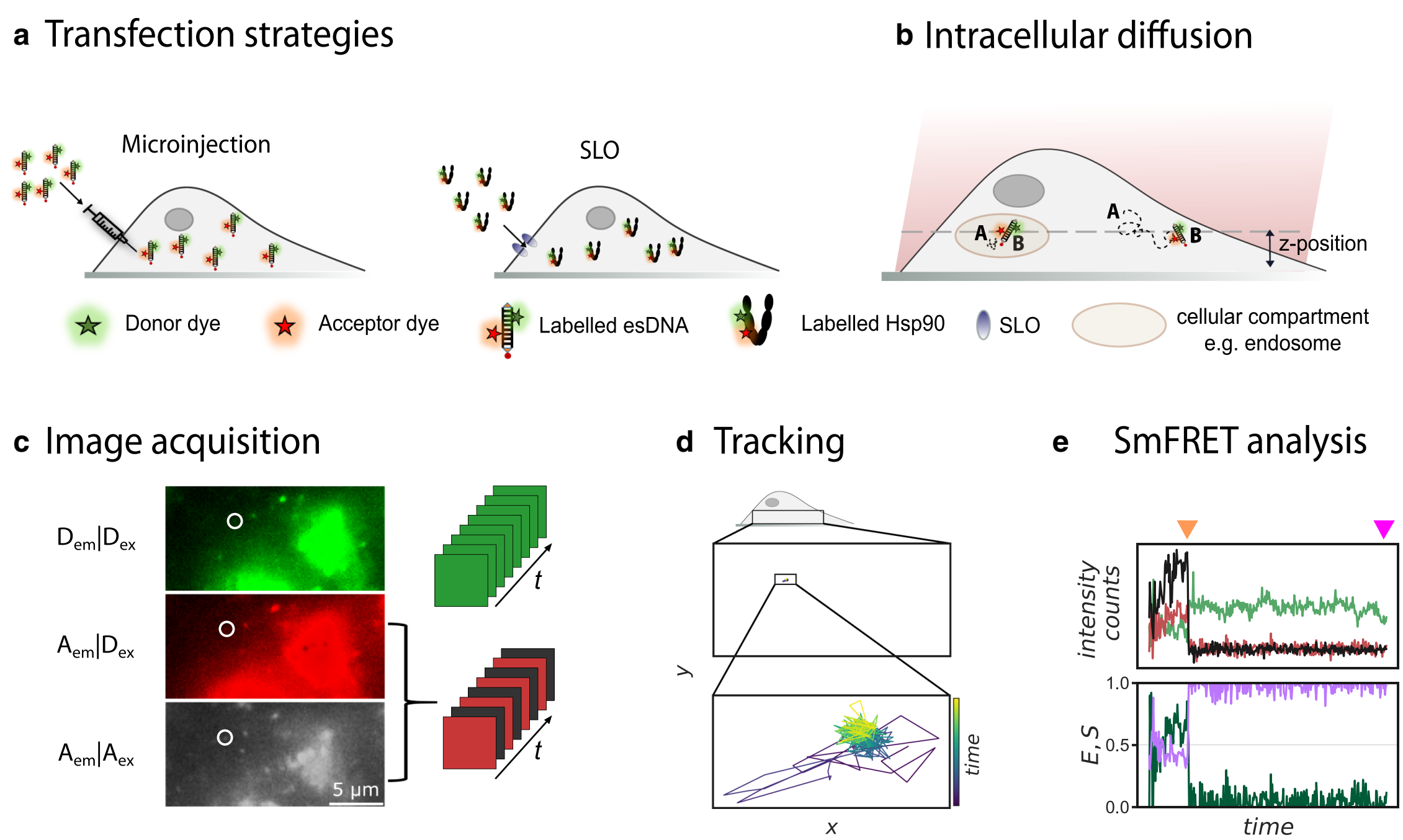

FIGURE 1 FRET-TTB workflow. (*a*) Schematic representation of microinjection (*left*) and streptolysin-O transfection (*right*) of living HeLa cells. Tested biomolecules are end-sealed DNA and the protein Hsp90_E409C_S601C, double labeled with Lumidyne dyes LD555 (donor) and LD655 (acceptor). (*b*) Labeled biomolecules are tracked with a custom-built HILO microscope in alternating laser excitation (ALEX) mode with integration times of 70 ms. (*c*) One frame of a SLO-transfected Hsp90 sample, demonstrating the ALEX excitation-emission strategy to obtain the three spatially resolved fluorescence signals: $I_{Dem|Dex}$, $I_{Aem|Dex}$, and $I_{Aem|Aex}$. (*d*) Example tracks from an Hsp90 protein, SLO-transfected in a HeLa cell and tracked for 62 s. (*e*) Example trace from the same protein showing $I_{Dem|Dex}$ in green, $I_{Aem|Dex}$ in red, and $I_{Aem|Aex}$ in black. The orange and pink arrowheads indicate acceptor and donor bleaching, respectively.

For details on the microinjection experiments see Note S4. SLO transfection was done according to (17). HeLa cells were seeded in 8-well dishes 1 day before the experiment. The cell number was adjusted to achieve 90–100% confluency on the day of the experiment. The samples were diluted with degassed Tyrode's buffer (140 mM NaCl, 5 mM KCl, 1 mM $MgCl_2$, 10 mM HEPES, 10 mM glucose [pH 7.4]) to a final concentration of 400 nM. For SLO activation, SLO (25,000 U/mL) and TCEP (final concentration 10 mM) were mixed and incubated for 20 min at 37°C. For the transfection, the cells were first washed with DPBS and then incubated with diluted SLO solution (125 U/mL in DPBS 1 mM $MgCl_2$) at 37°C for 10 min. During incubation, SLO inserts into the plasma membrane and assembles into large pores. After incubation, the cells were gently washed with DPBS 1 mM $MgCl_2$ and then incubated with the prepared sample on ice for 5 min. Then, the cells were washed with Tyrode's buffer. For recovery, the cells were then incubated for 20 min with recovery medium (DMEM, 10% FBS, GlutaMAX [1×], sodium pyruvate [1×], 2 mM ATP) at 37°C. To minimize background fluorescence, the cells were then washed with DPBS and finally imaged in live-cell imaging medium. All buffers and media were prewarmed at 37°C.

HILO measurements

Live-cell experiments were conducted on a custom-built HILO microscope as described before (28) and detailed in Note S5 and Fig. S2. The observation depth could be controlled independently of the excitation beam, as described before (29). The sample was mounted in a temperature-controllable chamber that guaranteed a constant temperature of 25°C during the measurements. Four excitation lasers were included and ALEX in green (553 nm, 10 mW) and red (637 nm, 11 mW) was used. The ALEX strategy was crucial to access single-molecule stoichiometry information and to detect photophysics of dyes in cellula, which otherwise might be misinterpreted as dynamics. Integration and read out times were set to 70 and 20 ms, respectively (180 ms per ALEX cycle). Fluorescence was recorded with two separate EMCCD cameras. Images were acquired with 256 × 122 pixels and binning of 2, resulting in a pixel size of 160 × 160 nm.

Confocal measurements

Single-molecule fluorescence experiments for Hsp90 in vitro were conducted at 25°C on a custom-built confocal setup as described before (30). Green (532 nm, LDH-D-FA-530L, PicoQuant, Berlin, Germany) and red laser light (640 nm, LDH-D-C-640, PicoQuant) were used to excite donor and acceptor dyes in pulsed interleaved excitation mode (31) with an repetition rate of 20 MHz. The laser power is 317 μW for green and 114 μW for red, respectively, and was measured before the objective. Before focusing on the sample by a 60× water immersion objective (CFI Plan Apo VC 60XC/1.2 WI, Nikon, Tokyo, Japan), both beams were polarized and superimposed by a dichroic mirror (F43-537 laser beam splitter z 532 RDC, AHF, Tübingen, Germany). Another dichroic mirror (F53-534 Dual Line beam splitter z 532/633, AHF) separates excitation and emission light. In the emission path a third dichroic mirror (F33-647 laser beam splitter 640 DCXR, AHF) separated donor fluorescence from acceptor fluorescence. Pinholes (50 μm diameter) filtered off-focus light. Before detection, polarizing beam splitters separated parallel and perpendicular polarized light. Donor and acceptor emission was detected after passing IR-filters (C-HSP750-25, LaserComponents, Olching, Germany) by single-photon detectors (two SPCM-AQRH-14-TR, Excelitas, and two PDM series APDs, Micro Photon Devices, Bolzano, Italy).

The measurements were performed in SiPEG surface-passivated chambers. Therefore, high-precision coverslips (Carl Roth, Karlsruhe, Germany; 24 nm × 60 mm (170 ± 5 μm)) were cleaned by sonication in 2% Hellmanex (Hellma Analytics, Müllheim, Germany), ultra-pure water, and isopropanol/water mixture (1:3) to dissolve impurities. Remaining droplets were removed with nitrogen. The slide surface was activated by low-pressure plasma (Tetra 30,

Diener Electronics, Ebhausen, Germany) performing subsequently an O_2 and air plasma process. The plasma process was directly followed by treating the activated coverslips with methoxy-silane-polyethylenglycol (mSiPEG) solution (5 kDa, Rapp Polymere, Tübingen, Germany) with a concentration of 30 mg/mL. Unbound mSiPEG was washed away with pure water. The cavity for the single-molecule experiments were formed by a two-component silicon polymer (Microset 101, Microset Products, Hinckley, UK). Before measurement, the chamber was preincubated with BSA (0.5 mg/mL) for about 20 min to prevent proteins from sticking.

The monomer-exchanged sample was diluted in degassed Tyrode's buffer to a final concentration of 200 pM and incubated for about 6 h with 2 mM AMP-PNP. AMP-PNP was used to populate the closed conformation. We chose AMP-PNP, because recent cryo-EM structures of Hsp90 complexes show Hsp90 in an N-terminal more closed conformation (32,33), which can be mimicked in vitro by adding AMP-PNP. Microtimes and macrotimes of detected photons in each detection channel were recorded for 3600 s in T3 mode using time-correlated single-photon counting (HydraHarp400, PicoQuant), with temporal resolutions of 16 ps and 50 ns, respectively.

Data analysis

Single-molecule experiments with HILO microscopy

As the transfected molecules can freely diffuse within the cytosol, tracking of the molecules must precede the actual smFRET data analysis. Localization and tracking of single particles was performed with a python-based software with Jupyter notebook interfaces, previously described in (34,35). Analysis of the fluorescence signals involved dual-channel tracking, i.e., co-tracking of molecules by first adding the signal intensity in the $Dem|Dex$ and $Aem|Dex$ channels and then co-tracking with the $Aem|Aex$ channel. Here, $Dem|Dex$ is donor emission upon donor excitation, $Aem|Dex$ is acceptor emission upon donor excitation, and $Aem|Aex$ is acceptor emission upon acceptor excitation. This methodology enables accurate tracking even when conformational changes and therefore changes in FRET efficiency occur (35). Upon tracking, the fluorescence intensity over time is extracted in the three channels $Dem|Dex$, $Aem|Dex$, and $Aem|Aex$ for each particle. With a generic user interface each recognized particle can be inspected. Based on fluorescence time traces, efficiency vs stoichiometry (ES) plots and the original movie a first manual selection of particles can be done. Only particles that show single-molecule features, such as single-step dye bleaching, extinction of the FRET signal upon bleaching of the first dye, and anticorrelated signal upon acceptor bleaching, are selected. Traces with multiple dyes are not further analyzed. The selected traces are then saved in each one txt file. The txt files are imported into Igor Pro for further smFRET analysis as, e.g., described in (36). Importantly, in Igor Pro the traces are manually segmented in three parts: 1) before bleaching, when both dyes are present and FRET can happen, 2) after the first dye bleached and only one dye shows a signal, and 3) after the second dye bleached and only background signal remains. For further FRET analysis only the first part is used while parts two and three can be used for data correction.

The FRET efficiency E is calculated from the fluorescence intensities of the dyes and the correction factors with Eq. 1 for each point in time (25). The correction factors were determined as detailed in Note S5.

$$E = \frac{I_{\mathrm{Aem|Dex}} - \alpha I_{\mathrm{Dem|Dex}} - \delta I_{\mathrm{Aem|Aex}}}{\gamma I_{\mathrm{Dem|Dex}} + I_{\mathrm{Aem|Dex}} - \alpha I_{\mathrm{Dem|Dex}} - \delta I_{\mathrm{Aem|Aex}}} \quad (1)$$

The stoichiometry S is calculated accordingly with Eq. 2.

$$S = \frac{\gamma I_{\mathrm{Dem|Dex}} + I_{Aem|Dex} - \alpha I_{Dem|Dex} - \delta I_{Aem|Aem}}{\gamma I_{Dem|Dex} + I_{Aem|Dex} - \alpha I_{\mathrm{Dem|Dex}} - \delta I_{Aem|Dex} + \beta I_{Aem|Aem}} \quad (2)$$

All FRET efficiency data are cumulated in histograms for a statistical analysis of the efficiency distributions. The correction factors α (cross talk), β (differences in absorption cross section and excitation powers of donor and acceptor fluorophores, respectively), γ (differences in quantum yield and detection efficiency), and δ (direct acceptor excitation) are given in Table S6. In Figs. 2 and 3 we show example traces of raw (uncorrected) data, while for the quantitative analysis in Fig. 4 we use the data corrected with the parameters given in Table S6.

Single-molecule experiments with confocal microscopy

The analysis of the confocal data was done using the Wolfram Mathematica package Fretica (https://schuler.bioc.uzh.ch/programs) (37). The "route correction matrix" corrects the number of photons for each detection channel. The route correction matrix was set by applying the correction factors according to the benchmark study (25). The G-factor was assumed to be 1. Using the ΔT burst search algorithm with dual-channel burst search (Fretica) with parameters $\Delta T = 150\ \mu s$ and $N_{max} = 100{,}000$, N_{min} was determined from the mean photon rate per second of the calculated background signal. The FRET efficiency E and the stoichiometry S were calculated for each identified single-molecule event according to Eqs. 1 and 2, correction factors are given in Table S6. Only single-molecule FRET events were taken into account in the transfer efficiency E histogram in Fig. 4 *a*. Five Gaussian shapes with a constrained width of 0.09 were assumed as the fitting model. Please note that the population highlighted in gray is most likely caused by the abundant donor-only population mainly caused by single- or overlabeled Hsp90 proteins.

RESULTS AND DISCUSSION

Benchmarking in cellula smFRET with end-sealed DNA

As cells inherently show high, inhomogeneous autofluorescence, we first wanted to understand whether the signal/noise ratio (SNR) of live-cell fluorescence time traces is sufficiently high to detect single-molecule FRET and to what extent the determined FRET efficiencies match with in vitro data. Therefore, we tested a stable and easy-to-transfect DNA sample with known dye distance, previously used for smFRET benchmark studies (25), labeled with Lumidyne dyes LD555 and LD655. We compared the quality of the fluorescence time traces as well as the obtained peak FRET efficiencies in three different environments with increasing complexity: in vitro, in fixed cells, and in living cells (Fig. 2, *top panel*).

In vitro, a high SNR with clear acceptor and donor bleaching can be obtained (Fig. 2 *a*). Because the molecules are immobilized, they can be traced for extended periods of more than a minute. The average FRET efficiency of 183 traces is $E = 0.80$, which replicates the values determined before (25). In both, fixed and living cells, the SNR substantially decreases, leading to a reduced number of clearly identifiable single molecules. This is because fluctuations in background noise during the molecules' diffusion are superimposed on the fluorophore signal. Nonetheless, a high SNR and clear bleaching steps can be obtained for a subset of traces. A total of 24 traces was obtained from 9 fixed cells while 26 traces were found in 52 living cells. Examples are shown in Fig. 2,

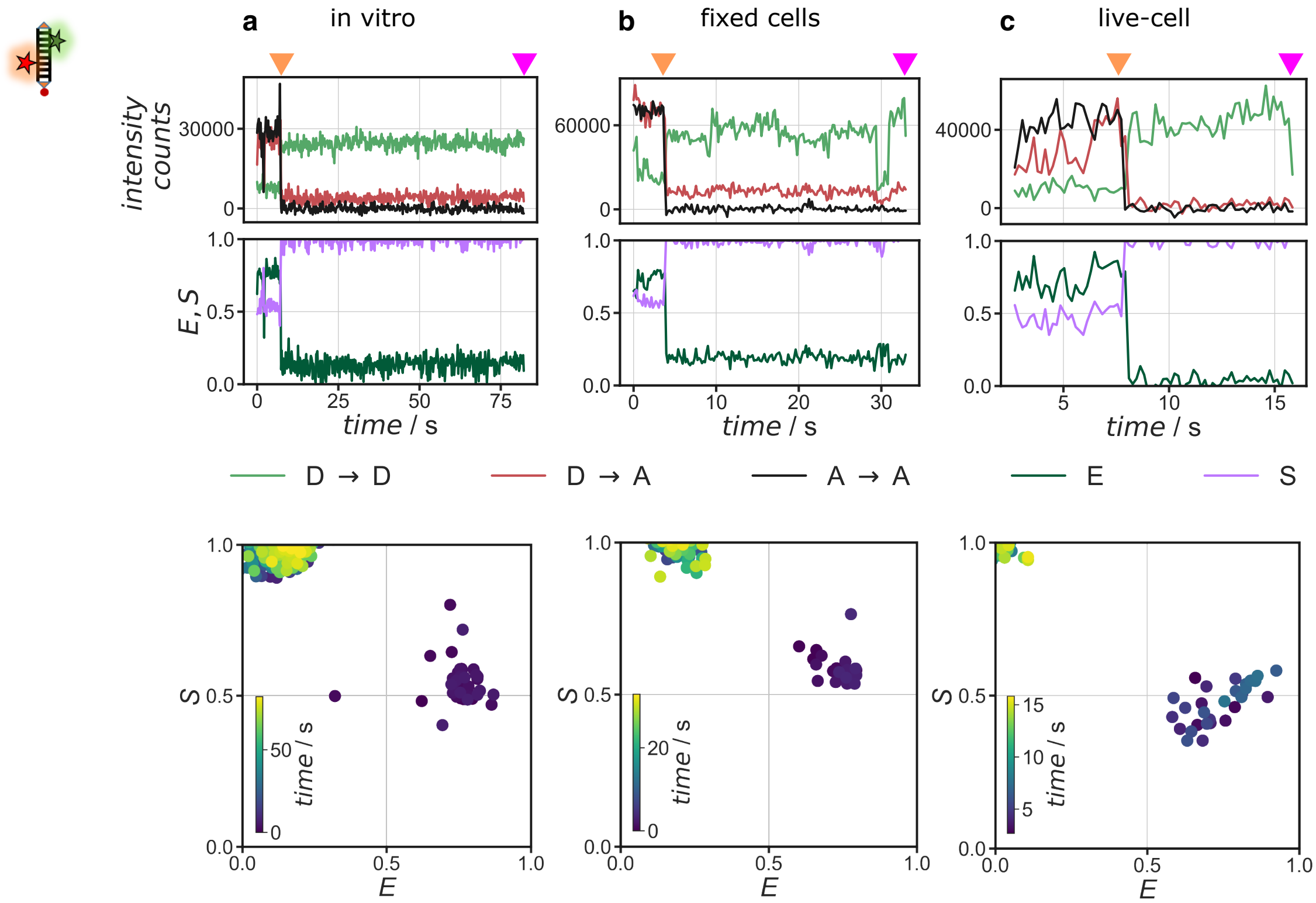

FIGURE 2 Comparison of in vitro and in cellula smFRET data for end-sealed DNA. Example traces of (*a*) an in vitro measurement (total 183 traces in 15 ROIs), (*b*) a fixed-cell measurement after microinjection (total 24 traces in 9 cells), and (*c*) a live-cell experiment after SLO transfection (26 traces in 52 cells). Intensities $I_{\mathrm{Dem|Dex}}$ (*green*), $I_{\mathrm{Aem|Dex}}$ (*red*), and $I_{\mathrm{Aem|Aex}}$ (*black*) from tracked single molecules as well as their FRET efficiency (*dark green*) and stoichiometry (*violet*) against time. Orange and pink arrows indicate acceptor and donor bleaching, respectively. The respective raw FRET efficiency (E) versus stoichiometry (S) plots are shown at the bottom. For additional traces and E versus S plots see Note S7.

b and *c*. With average values of 0.63 and 0.61 for fixed and live cells, respectively, the FRET efficiency decreases by approximately 25% compared with the efficiency determined in vitro. Considering the complex nature of the cellular environment, this behavior is not unexpected and may arise from multiple factors like dynamic quenching of the fluorophores caused by collisions with small molecules (e.g., oxygen) or by static quenching through interactions of the fluorophores with proteins binding to the DNA. Such effects are much more prominent in the macromolecular crowded cytosol and reduce the fluorescence intensity and therefore likely the FRET efficiency.

The unambiguous smFRET signal (single donor and acceptor bleaching steps and anticorrelated behavior) proves that, with our FRET-TTB strategy, we are able to 1) track single molecules, 2) extract fluorescence intensities, and 3) determine smFRET efficiencies and stoichiometries of labeled molecules in fixed and living cells.

For further experiments with protein samples, we focused on the transfection with SLO. Microinjection is a low-throughput method and special instrumentation as well as extensive training are needed for successful transfections. This currently renders the method less efficient and less suited for a broad application.

In cellula smFRET with the protein Hsp90

Next, we show that the transfection of HeLa cells also works with a dimer of Hsp90. Generally, complex multidimensional motions within proteins lead to a less-stable fluorescence signal when compared with the rather stable, linear, short DNA oligos, used as a FRET standard sample. While immobilization and background correction help maintain a high SNR in vitro, it is not possible for freely diffusing biomolecules in living cells.

Consequently, many of the traces become difficult to interpret, but by acquisition of large data sets, we were able to obtain traces with a sufficient SNR for smFRET analysis. Examples are shown in Fig. 3, *b* and *c* (*left panel*). Clear single-step bleaching of the acceptor indicates that single molecules are observed, while the anticorrelated increase in donor intensity is a strong indicator for FRET. Furthermore, the stoichiometry (S) versus FRET efficiency (E) plots (Fig. 3, *b* and *c*, *middle panel*) clearly separate a donor-only population at $S \approx 1$ and $E \approx 0$ and a FRET population at $S \approx 0.5$.

Besides smFRET information, our analysis also provides access to tracking data, enabling us to monitor the intracellular movement of molecules. The two particles, shown in Fig. 3, were tracked for about 62 s (792 frames) and 29 s (326 frames),

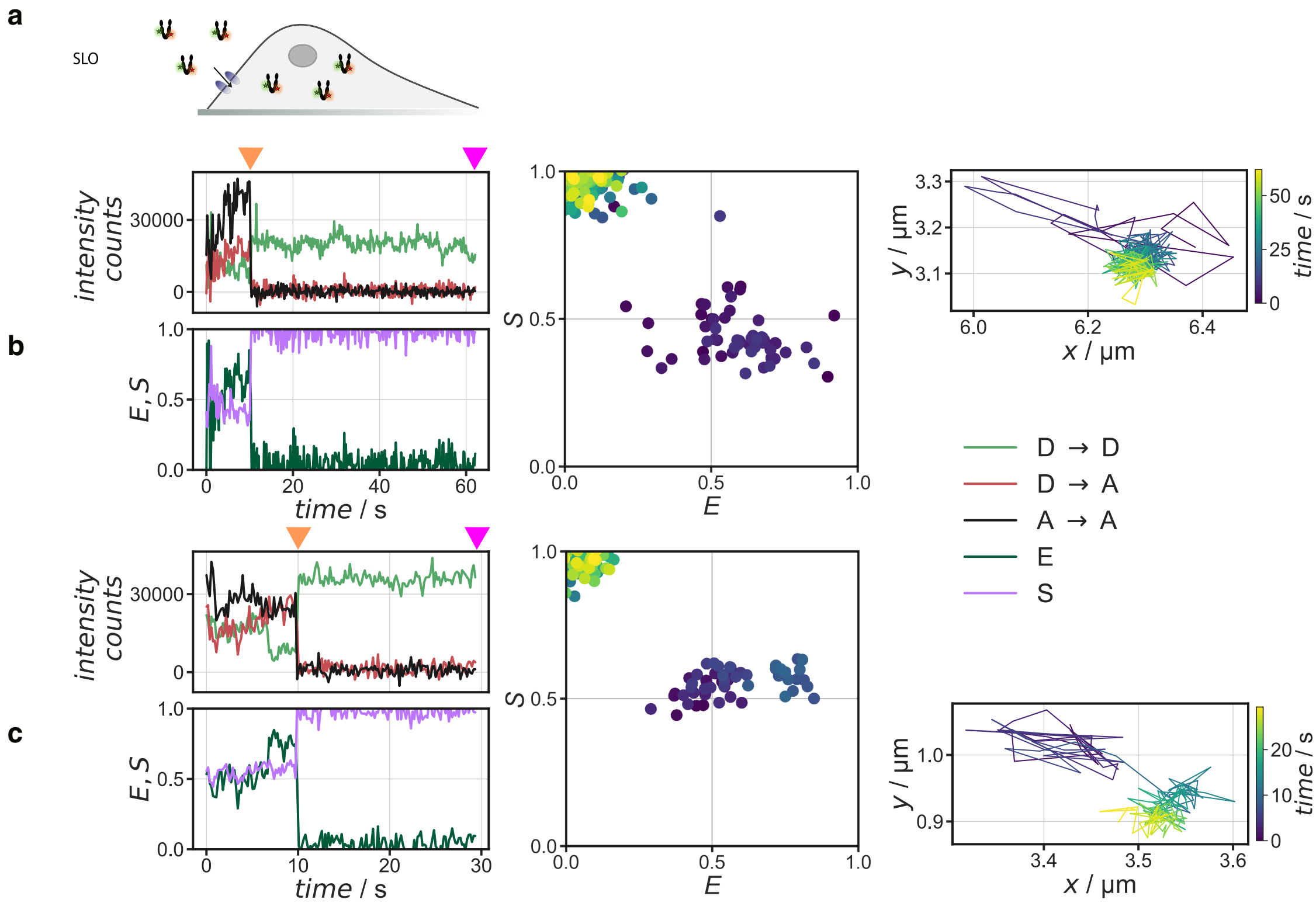

FIGURE 3 Live-cell single-molecule FRET data with Hsp90_409_601. (*a*) Schematics of the SLO-mediated transfection of HeLa cells with double-labeled Hsp90. (*b* and *c*) Two examples of fluorescence intensity traces, their corresponding stoichiometry versus FRET efficiency plots and tracks (from *left* to *right*). In total, 51 traces from 100 cells were obtained.

respectively. From the corresponding trajectories (see Fig. 3) diffusion coefficients of $D = (5.6 \pm 22) \times 10^{-6}\ \mu m^2/s$ and $D = (1.1 \pm 0.47) \times 10^{-4}\ \mu m^2/s$ were determined. Diffusion coefficients previously measured for various proteins in the cytosol of living prokaryotic and eukaryotic cells typically range from 10^{-2} to $10^{2}\ \mu m^2/s$ (38–40), which is several orders of magnitude higher than the coefficients determined in our study. This indicates that, instead of free diffusion largely nondiffusive, stagnant states have been observed. As Hsp90 does not have any anchor to restrict movement in cellula, it is likely that the observed molecules are trapped within cellular compartments such as endosomes or lysosomes. This work lays the groundwork for a more sophisticated analysis to, e.g., extract diffusion states (41). By combining tracking and smFRET information, it will be possible to address many questions, e.g., whether the subcellular localization in different compartments influences a protein's conformational state.

Comparison of Hsp90's conformational states in vitro and in living cells

Screening of 100 individual cells, transfected with Hsp90_409_601 resulted in 51 smFRET traces. Fig. 4 shows the distribution of all calculated FRET efficiencies (Fig. 4 *b*) alongside in vitro data of the same construct in the presence of 2 mM AMP-PNP (Fig. 4 *a*). We aimed to produce Hsp90 dimers with exactly one donor and one acceptor dye, both bound to a single monomer. With this, a FRET efficiency peak between $E = 0.354$ and $E = 0.435$, the respective calculated FRET efficiencies for the open and closed conformations, was expected (see Note S1 for details). Indeed, with 23% relative occurrence in vitro and 42% in cellula, both FRET efficiency distributions show large contributions at the expected FRET efficiency of around 0.4. But there are several additional peaks. These other FRET efficiencies are likely caused by suboptimal monomer exchange. Statistically 95% of the Hsp90 dimers are expected to carry a maximum of two dyes in one monomer and none in the second monomer. Nevertheless, when the monomers are not fully exchanged and dyes are bound to both monomers, a multitude of FRET efficiencies are possible (see Tables S1 and S2 for all combinations). For a quantitative comparison, we chose to fit four Gaussians to the FRET efficiency distributions in vivo, whereas an additional one was included in vitro to account for artifacts most likely from donor-only labeled proteins. Comparing these four most pronounced contributions (see Table 1), we see remarkable agreement for the peak positions.

Minor differences in peak positions and size are expected, given the crowded environment of the cell and the numerous specific interactions of Hsp90 with cochaperones and

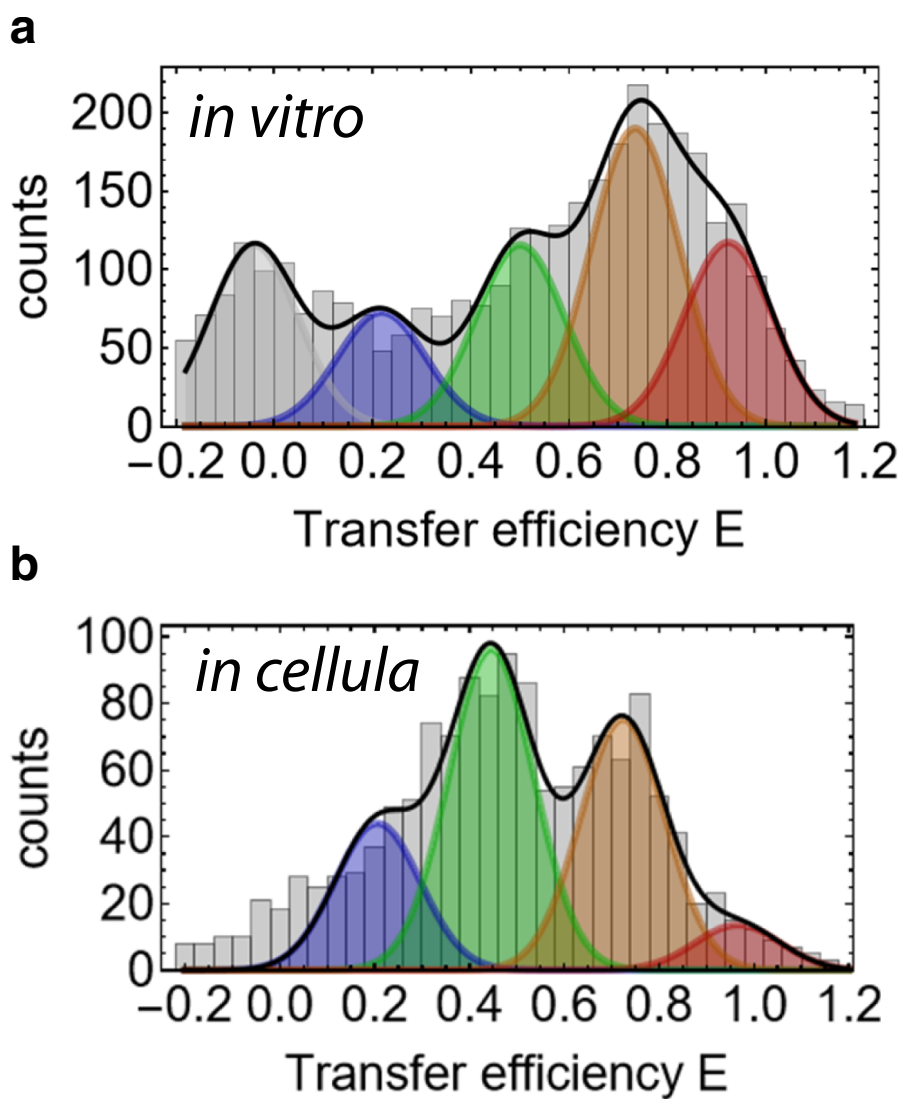

FIGURE 4 FRET efficiency distributions of Hsp90_409_601 in vitro and in cellula. (*a*) In vitro smFRET-PIE data recorded on a confocal microscope for 1 h, measured in presence of AMP-PNP. (*b*) In cellula data recorded on a HILO microscope from a total of 100 cells and 51 single-molecule traces. Four Gaussians were fit to the data. For the confocal data an additional very low FRET Gaussian was used (*gray*), to account for bursts most likely from single labeled proteins (donor only).

clients. Some differences in occurrence might also be due to labeling deviations in different sample batches. Altogether, our results underline the suitability of the presented live-cell FRET-TTB approach to investigate conformational state changes of proteins in their native environment. We demonstrated that different FRET states can be resolved and statistically robust results obtained.

CONCLUSION

Our approach provides a controlled and effective means of observing and understanding the multidimensional conformations of biomolecules within living cells. This is essential for gaining insight into the functions of proteins that likely vary in changing environments and cellular compartments. With our home-built HILO setup, we were able to observe and track single DNA and protein molecules inside the cytoplasm and likely other compartments of living cells. ALEX allowed us to obtain dye stoichiometry information, which is important for quality control, in particular to detect dye photophysics. Stable smFRET traces with good SNR obtained from end-sealed DNA validated our method. Extending the measurements to the more challenging protein samples demonstrated the power of this technique. Clear single-molecule traces as well as a comparison of the FRET efficiency distribution with in vitro data obtained from confocal smFRET experiments proved the method successful.

In conclusion, we have presented a method to investigate single biopolymers in living cells based on single-molecule FRET-TTB. This allowed us to track molecules for up to 90 s and determine smFRET for up to 20 s and therefore to step into long-term smFRET and stoichiometry analysis using fluorescence intensity information. In the cytosol of cells, this was not possible before due to technical limitations and short observation times. Furthermore, the developed method is not restricted to a specific area of the cell and is therefore a valuable tool for investigating protein plasticity and localization-dependent function of proteins. The precise control of the protein before transfection will enable us to quantify the response of biomolecules to different cellular environments or compartments in an unprecedented way.

TABLE 1 Comparison of the in vitro and in cellula FRET efficiency distributions for yHsp90_E409C_S601C

	in vitro		in cellula	
	E	Occ. (%)	*E*	Occ. (%)
Low FRET	0.21	15	0.21	19
Mid FRET 1	0.50	23	0.45	42
Mid FRET 2	0.73	38	0.72	33
High FRET	0.92	24	0.97	6

E, mean efficiency of the Gauss fits; occ., relative abundance of FRET efficiencies within the Gauss fits.

ACKNOWLEDGMENTS

This work is dedicated to Prof. Dr. Erich Sackmann, an outstanding scientist, teacher, and personality, whose inspiration had a lasting impact on this and many other studies. The work was supported by the Deutsche Forschungsgemeinschaft (DFG) under Germany's Excellence Strategy (CIBSS EXC-2189 project ID 390939984) and the SFB1381 (project ID 403222702) and by the European Research Council through ERC grant agreement no. 681891. We thank Dr. Fernando Aprile-Garcia and Dr. Ritwick Sawarkar for help with setting up the cell culture and the viability assays, we thank Dr. Bianca Hermann, Jolanta Vorreiter, and Michael Witt for their help in protein production of Hsp90 variants, we thank Marianne Birkle for help with the cell cultures, we thank Prof. Dr. Ben Schuler for aiding us in running our initial tests of Hsp90 on their confocal injection setup, we thank Dr. Bismark Appiah for providing his expertise in handling the Femtojet II injector and micromanipulator for cell experiments, and we thank Dr. Yasser Gidi and Dr. Liwei Zheng for helpful discussions. ChatGPT, developed by OpenAI, assisted by providing some suggestions for language improvements in some paragraphs.

AUTHOR CONTRIBUTIONS

A.A. and T.H. designed the research. A.A. performed the microinjection experiments. A.E. performed the SLO experiments and data analysis. V.F. performed the confocal microscopy experiments and data analysis. P.W. and A.A. performed the initial tests with SLO. L.S. and G.J.S. provided and optimized the dual-channel tracking software with the help of A.A. and A.E. A.A., T.H., and C.L. designed and co-built the HILO setup. A.A., A.E., and T.H. wrote the manuscript with the help of all authors. All authors approved the final version of the manuscript.

DECLARATION OF INTERESTS

The authors declare no conflict of interest.

SUPPORTING MATERIAL

Supporting material can be found online at https://doi.org/10.1016/j.bpj.2025.09.024. Raw data will be uploaded on Zenodo.org.

REFERENCES

1. Götz, M., A. Barth, …, S. Schmid. 2022. A blind benchmark of analysis tools to infer kinetic rate constants from single-molecule fret trajectories. *Nat. Commun.* 13:5402. https://doi.org/10.1038/s41467-022-33023-3.
2. Asher, W. B., P. Geggier, …, J. A. Javitch. 2021. Single-molecule fret imaging of gpcr dimers in living cells. *Nat. Methods.* 18:397–405. https://doi.org/10.1038/s41592-021-01104-4.
3. Sackmann, E., and M. Tanaka. 2021. Critical role of lipid membranes in polarization and migration of cells: a biophysical view. *Biophys. Rev.* 13:123–138. https://doi.org/10.1007/s12551-021-00781-1.
4. König, I., A. Zarrine-Afsar, …, B. Schuler. 2015. Single-molecule spectroscopy of protein conformational dynamics in live eukaryotic cells. *Nat. Methods.* 12:773–779. https://doi.org/10.1038/nmeth.3456.
5. Ha, T., J. Fei, …, S. Yeou. 2024. Single-molecule fluorescence resonance energy transfer (smfret). *Nat. Rev. Methods Primers.* 4:21. https://doi.org/10.1038/s43586-024-00298-3.
6. Sankaran, J., and T. Wohland. 2023. Current capabilities and future perspectives of fcs: super-resolution microscopy, machine learning, and in vivo applications. *Commun. Biol.* 6:699. https://doi.org/10.1038/s42003-023-05069-6.
7. Tokunaga, M., N. Imamoto, and K. Sakata-Sogawa. 2008. Highly inclined thin illumination enables clear single-molecule imaging in cells. *Nat. Methods.* 5:159–161.
8. Göhring, J., F. Kellner, …, G. J. Schütz. 2021. Temporal analysis of t-cell receptor-imposed forces via quantitative single molecule fret measurements. *Nat. Commun.* 12:1–14. https://doi.org/10.1038/s41467-021-21224-1.
9. Kapanidis, A. N., N. K. Lee, …, S. Weiss. 2004. Fluorescence-aided molecule sorting: Analysis of structure and interactions by alternating-laser excitation of single molecules. *Proc. Natl. Acad. Sci. USA.* 101:8936–8941. https://doi.org/10.1073/pnas.0401690101.
10. Kapanidis, A. N., T. A. Laurence, …, S. Weiss. 2005. Alternating-laser excitation of single molecules. *Acc. Chem. Res.* 38:523–533. https://doi.org/10.1021/ar0401348.
11. Nelson, E. M., V. Kurz, …, G. Timp. 2012. Using a nanopore for single molecule detection and single cell transfection. *Analyst.* 137:3020–3027. https://doi.org/10.1039/C2AN35021B.
12. Fu, A., R. Tang, …, V. M. Rotello. 2014. Promises and pitfalls of intracellular delivery of proteins. *Bioconjug. Chem.* 25:1602–1608. https://doi.org/10.1021/bc500245j.
13. Yu, J. 2016. Single-molecule studies in live cells. *Annu. Rev. Phys. Chem.* 67:565–585. https://doi.org/10.1146/annurev-physchem-040215-112451.
14. Walev, I., S. C. Bhakdi, …, S. Bhakdi. 2001. Delivery of proteins into living cells by reversible membrane permeabilization with streptolysin-o. *Proc. Natl. Acad. Sci. USA.* 98:3185–3190. https://doi.org/10.1073/pnas.051551098.
15. Corrotte, M., M. C. Fernandes, …, N. W. Andrews. 2012. Toxin pores endocytosed during plasma membrane repair traffic into the lumen of mvbs for degradation. *Traffic.* 13:483–494. https://doi.org/10.1111/j.1600-0854.2012.01319.x.
16. Stewart, S. E., M. E. D'Angelo, …, P. I. Bird. 2015. Assembly of streptolysin o pores assessed by quartz crystal microbalance and atomic force microscopy provides evidence for the formation of anchored but incomplete oligomers. *Biochim. Biophys. Acta Biomembr.* 1848:115–126. https://doi.org/10.1016/j.bbamem.2014.10.002.
17. Teng, K. W., Y. Ishitsuka, …, P. R. Selvin. 2016. Labeling proteins inside living cells using external fluorophores for microscopy. *eLife.* 5:e20378. https://doi.org/10.7554/eLife.20322.
18. Shimizu, N., F. Kamezaki, and S. Shigematsu. 2005. Tracking of microinjected dna in live cells reveals the intracellular behavior and elimination of extrachromosomal genetic material. *Nucleic Acids Res.* 33:6296–6307. https://doi.org/10.1093/nar/gki939.
19. Wegmann, S., B. Eftekharzadeh, …, B. T. Hyman. 2018. Tau protein liquid–liquid phase separation can initiate tau aggregation. *EMBO J.* 37:e98049. https://doi.org/10.15252/embj.201798049.
20. Mishra, P., and D. N. A. Bolon. 2014. Designed hsp90 heterodimers reveal asymmetric atpase-driven mechanism in vivo. *Mol. Cell.* 53:344–350. https://doi.org/10.1016/j.molcel.2013.12.020.
21. Li, J., J. Soroka, and J. Buchner. 2012. The hsp90 chaperone machinery: Conformational dynamics and regulation by co-chaperones. *Biochim. Biophys. Acta.* 1823:624–635. https://doi.org/10.1016/j.bbamcr.2011.09.010.
22. Michael, M., M. Hessling, …, T. Hugel. 2009. The large conformational changes of hsp90 are only weakly coupled to atp hydrolysis. *Nat. Struct. Mol. Biol.* 16:281–286. https://doi.org/10.1038/nsmb.1569.
23. Schmid, S., M. Götz, and T. Hugel. 2018. Effects of inhibitors on hsp90's conformational dynamics, cochaperone and client interactions. *ChemPhysChem.* 19:1716–1721. https://doi.org/10.1002/cphc.201701379.
24. Silbermann, L. M., B. Vermeer, …, K. Tych. 2024. The known unknowns of the hsp90 chaperone. *eLife.* 13:e102666. Published 2024 Dec 31. https://doi.org/10.7554/eLife.102666.
25. Hellenkamp, B., S. Schmid, …, T. Hugel. 2018. Precision and accuracy of single-molecule fret measurements—a multi-laboratory benchmark study. *Nat. Methods.* 15:669–676. https://doi.org/10.1038/s41592-018-0139-8.
26. Hellenkamp, B., P. Wortmann, …, T. Hugel. 2017. Multidomain structure and correlated dynamics determined by self-consistent fret networks. *Nat. Methods.* 14:174–180. https://doi.org/10.1038/nmeth.4081.
27. Kalinin, S., T. Peulen, …, C. A. M. Seidel. 2012. A toolkit and benchmark study for fret-restrained high-precision structural modeling. *Nat. Methods.* 9:1218–1225. https://doi.org/10.1038/nmeth.2222.
28. Lan, C., J. Kim, …, T. Hugel. 2023. Quantitative real-time in-cell imaging reveals heterogeneous clusters of proteins prior to condensation. *Nat. Commun.* 14:4831. https://doi.org/10.1038/s41467-023-40540-2.
29. Kuchler, O., J. Gerlach, …, B. Knöll. 2022. Single-molecule tracking (smt) and localization of srf and mrtf transcription factors during neuronal stimulation and differentiation. *Open Biol.* 12:210383. https://doi.org/10.1098/rsob.210383.
30. Sohmen, B., C. Beck, …, T. Hugel. 2023. The onset of molecule-spanning dynamics in heat shock protein hsp90. *Adv. Sci.* 10:2304262. https://doi.org/10.1002/advs.202304262.
31. Müller, B. K., E. Zaychikov, …, D. C. Lamb. 2005. Pulsed interleaved excitation. *Biophys. J.* 89:3508–3522. https://doi.org/10.1529/biophysj.105.064766.
32. Wang, R. Y. R., C. M. Noddings, …, D. A. Agard. 2022. Structure of hsp90–hsp70–hop–gr reveals the hsp90 client-loading mechanism. *Nature.* 601:460–464.
33. Noddings, C. M., R. Y. R. Wang, …, D. A. Agard. 2022. Structure of hsp90–p23–gr reveals the hsp90 client-remodelling mechanism. *Nature.* 601:465–469.
34. Schrangl, L., J. Göhring, …, G. J. Schütz. 2021. Automated two-dimensional spatiotemporal analysis of mobile single-molecule fret probes. *JoVE J.* 177:e63124.
35. Schrangl, L., J. Göhring, G. J. Schütz…, 2024. Measurement of forces acting on single t-cell receptors. *In* Methods in Molecular Biology in Imaging Cell Signaling, *2800*. C. Wuelfing and R. F. Murphy, eds. Springer, p. 147.

36. Gotz, M., P. Wortmann, …, T. Hugel. 2018. Using three-color single-molecule fret to study the correlation of protein interactions. *JoVE J.* 131:e56896. https://doi.org/10.3791/56896.

37. Nüesch, M. F., M. T. Ivanović, …, B. Schuler. 2022. Single-molecule spectroscopy of protein conformations in live eukaryotic cells. *J. Am. Chem. Soc.* 144:52–56. https://doi.org/10.1021/jacs.1c09387.

38. Kühn, T., T. O. Ihalainen, …, J. Timonen. 2011. Protein diffusion in mammalian cell cytoplasm. *PLoS One.* 6:e22962.

39. Mika, J. T., and B. Poolman. 2011. Macromolecule diffusion and confinement in prokaryotic cells. *Curr. Opin. Biotechnol.* 22:117–126.

40. Schavemaker, P. E., A. J. Boersma, and B. Poolman. 2018. How important is protein diffusion in prokaryotes? *Front. Mol. Biosci.* 5:93.

41. Rahm, J. V., S. Malkusch, …, M. Heilemann. 2021. Diffusion state transitions in single-particle trajectories of met receptor tyrosine kinase measured in live cells. *Front. Comput. Sci.* 3:757653. https://doi.org/10.3389/fcomp.2021.757653.

Biophysical Journal, Volume *125*

Supplemental information

Single-molecule FRET and tracking of transfected biomolecules in living cells

Abhinaya Anandamurugan, Antonia Eidloth, Veronika Frank, Philipp Wortmann, Lukas Schrangl, Chenyang Lan, Gerhard J. Schütz, and Thorsten Hugel

Supplementary Information for: Single-molecule FRET and Tracking of Transfected Biomolecules in Living Cells

Abhinaya Anandamurugan*,†1, Antonia Eidloth*1, Veronika Frank[1], Philipp Wortmann[1], Lukas Schrangl[3], Chenyang Lan[1], Gerhard J. Schütz[3], and Thorsten Hugel†1,2

[1]Institute of Physical Chemistry, University of Freiburg, Albertstr. 21, 79104 Freiburg, Germany
[2]Signalling research centers BIOSS and CIBSS, University of Freiburg, Germany
[3]Institute of Applied Physics, TU Wien, 1060 Vienna, Austria.

September 12, 2025

1. **Suppl. Note 1: FRET efficiency calculations** 2
2. **Suppl. Note 2: Protein production** 5
3. **Suppl. Note 3: Labelling of Biomolecules** 6
4. **Suppl. Note 4: Microinjection** 7
5. **Suppl. Note 5: Tracking Microscopy (HILO)** 9
6. **Suppl. Note 6: Data Analysis** 12
7. **Suppl. Note 7: Example data and S vs E plots** 14

*Equal contribution

†Corresponding authors: th@pc.uni-freiburg.de; abhinaya.anandamurugan@gmail.com

Supplementary Note1: AV and distance calculation

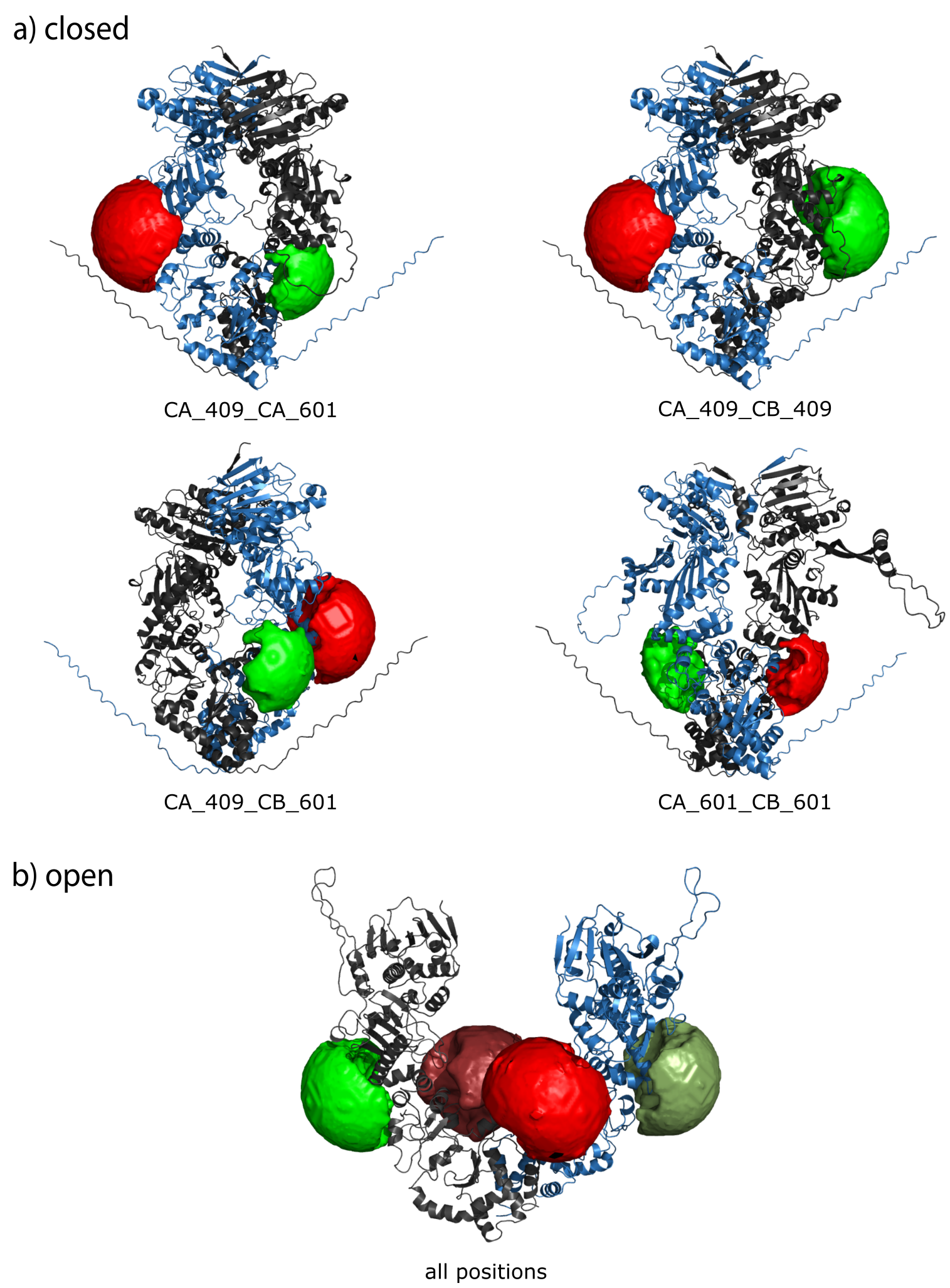

Figure 1: Some possible combinations of labeling positions for the cysteins at positions 409 and 601. Upper four figures based on an Alphafold2 structure for closed yeast Hsp90. Bottom based on the open structure of yeast Hsp90 [1]. Accessible volumes for donor and acceptor are based on FPS analysis [2]. They are depicted in green and red, respectively. CA: Chain A; CB: Chain B.

Accessible dye volume (AV) simulations and distance calculations were performed with the Lumidyne dye pair LD555 and LD655 (Lumidyne Tech). As the original crystal structure of Hsp90 (2cg9) does not fully resolve the C-terminal end, we used the Alphafold2 structure for closed yeast Hsp90. The open struc-

ture of Hsp90 (m-om4) was published before in [1]. Assuming that exactly two dyes are bound to Hsp90, the distances for all possible combinations of dye positions were calculated using the software FPS [2]. The AVs for various combinations are depicted in Suppl.Fig. 1 and the calculated distances and FRET efficiencies are summarized in Suppl. Table 1.

Table 1: Distances for different combinations of labeling positions for the closed Hsp90_409_601 dimer. Calculations were done with dye parameters for LD555 and LD655. Specifications of the two dyes (#1, #2): the dye-position (CA-chain A; CB-chain B) and the kind of dye (A-Acceptor; D-Donor); Rmp-distance between the mean dye positions; $\langle RDA \rangle E$-FRET averaged distance; E-FRET Efficiency.

#1	#2	*Rmp*	$\langle RDA \rangle$	*sigmaDA*	**E**	$\langle RDA \rangle E$
CA_409_A	CA_601_D	63,7	65,3	7	0,435	64,8
CA_409_A	CB_409_D	82,8	84,7	7,1	0,147	83,2
CA_409_A	CB_601_D	33,5	36,3	8,2	0,937	39,5
CA_409_D	CA_601_A	63,7	65,3	7	0,435	64,8
CA_409_D	CB_409_A	82,8	84,7	7,1	0,146	83,2
CA_409_D	CB_601_A	33,5	36,3	8,2	0,937	39,5
CA_601_A	CA_409_D	63,7	65,3	7	0,435	64,8
CA_601_A	CB_409_D	33,5	36,2	8,1	0,938	39,4
CA_601_A	CB_601_D	48,8	49,8	5,9	0,771	50,6
CA_601_D	CA_409_A	63,7	65,3	7	0,435	64,8
CA_601_D	CB_409_A	33,5	36,2	8,1	0,938	39,4
CA_601_D	CB_601_A	48,8	49,8	5,9	0,771	50,6
CB_409_A	CB_601_D	63,8	65,4	7	0,434	64,8
CB_409_A	CA_409_D	82,8	84,8	7,1	0,146	83,2
CB_409_A	CA_601_D	33,5	36,3	8,1	0,938	39,5
CB_409_D	CB_601_A	63,8	65,4	7	0,434	64,8
CB_409_D	CA_409_A	82,8	84,8	7,1	0,146	83,2
CB_409_D	CA_601_A	33,5	36,3	8,1	0,938	39,5
CB_601_A	CB_409_D	63,8	65,4	7	0,434	64,8
CB_601_A	CA_409_D	33,5	36,3	8,2	0,937	39,5
CB_601_A	CA_601_D	48,8	49,9	5,9	0,771	50,6
CB_601_D	CB_409_A	63,8	65,4	7	0,434	64,8
CB_601_D	CA_409_A	33,5	36,3	8,2	0,937	39,5
CB_601_D	CA_601_A	48,8	49,9	5,9	0,771	50,6

Table 2: Distances for different combinations of labeling positions for the open Hsp90_409_601 dimer. Calculations were done with dye parameters for LD555 and LD655. Specifications of the two dyes (#1, #2): the dye-position (CA-chain A; CB-chain B) and the kind of dye (A-Acceptor; D-Donor); Rmp-distance between the mean dye positions; $\langle RDA \rangle E$-FRET averaged distance; E-FRET Efficiency.

#1	#2	Rmp	$\langle RDA \rangle$	$sigmaDA$	**E**	$\langle RDA \rangle E$
CA_409_A	CA_601_D	68,1	70,2	9,9	0,354	68,6
CA_409_A	CB_409_D	111,9	113,4	7,8	0,029	111,5
CA_409_A	CB_601_D	47,5	50,6	8,7	0,742	52
CA_409_D	CA_601_A	68,1	70,2	9,9	0,354	68,5
CA_409_D	CB_409_A	111,9	113,3	7,8	0,029	111,5
CA_409_D	CB_601_A	47,5	50,6	8,7	0,742	52
CA_601_A	CA_409_D	68,1	70,2	9,9	0,354	68,5
CA_601_A	CB_409_D	47,1	50,3	8,8	0,747	51,8
CA_601_A	CB_601_D	33,8	37,4	12,2	0,9	43
CA_601_D	CA_409_A	68,1	70,2	9,9	0,354	68,5
CA_601_D	CB_409_A	47,1	50,3	8,8	0,747	51,8
CA_601_D	CB_601_A	33,8	37,4	12,2	0,9	43
CB_409_A	CB_601_D	67,8	69,8	9,8	0,359	68,3
CB_409_A	CA_409_D	111,9	113,3	7,8	0,029	111,5
CB_409_A	CA_601_D	47,1	50,3	8,8	0,748	51,7
CB_409_D	CB_601_A	67,8	69,9	9,8	0,359	68,3
CB_409_D	CA_409_A	111,9	113,3	7,8	0,029	111,5
CB_409_D	CA_601_A	47,1	50,3	8,8	0,748	51,7
CB_601_A	CB_409_D	67,8	69,9	9,8	0,36	68,2
CB_601_A	CA_409_D	47,5	50,6	8,7	0,742	52
CB_601_A	CA_601_D	33,8	37,4	12,2	0,9	43
CB_601_D	CB_409_A	67,8	69,9	9,8	0,359	68,3
CB_601_D	CA_409_A	47,5	50,6	8,7	0,742	52
CB_601_D	CA_601_A	33,8	37,4	12,2	0,9	43

Supplementary Note 2: Protein production

Hsp90z_Avi_409C_601C: Cysteine residues were introduced into the sequence of yeast Hsp90 via site-directed mutagenesis at positions 409 and 601 (Quick Change Lighting kit, Agilent). The cysteine modified Hsp90 was expressed with a pET28 vector. Additionally this protein has an Avi-tag, which leads to a biotinylation in the bacterial cell with the help of biotin ligase enzymes co-expressed in a pBirAcm (Avidity Nanomedicine, La Jolla, CA) plasmid. The cells were grown in LB media supplemented with 0.5% glucose and Kanamycin (100µg/ml) at 37 °C. At a OD of 0.7, the expression was induced by adding isopropyl β-D-1-thiogalactopyranoside (IPTG; Sigma; final concentration 1 M). Furthermore, biotin (prepared in 10 mM Bicine Buffer pH 8.3 (adjusted with 1 M KOH, filter sterilized)) was added to the medium with a final concentration of 50 μM. The cells were then harvested 4 hours after induction and pelleted by centrifugation at 6000 rpm. Cell pellets are stored at -20°C until protein purification. For cell lysis pellet was resuspended in NiNTA A buffer with 20 mM imidazole. Protease inhibitor 50x is added prior to cell disruption in a cell disruptor (I & L Biosystems GmbH). $MgCl_2$ (final concentration 2 mM) and 6 µl of Benzonase (EMD Millipore Corp, USA) are added to the cell lysate. It is then centrifuged for 1 h at 50,000g. The supernatant is then passed through a 0.45 filter (Merck Millipore millex), the filtrate is then collected and fed to the HisTrap. The filtrate collected is then fed to the sample pump for HisTrap1. The NiNTA HisTrap column is pre-washed and equilibrated with HisA1 buffer. The sample is loaded onto the column and then allowed to run through the HisTrap1. The bound fraction is collected. The outlet collected from HisTrap1 is fed again into the HisTrap2. The bound fraction is again collected and diluted so the total salt concentration is less than 30mM. This sample is then fed into HiTrapQ. The eluted fraction is then collected and then concentrated to less than 3 g/l and injected into the Size exclusion column 26/600. The final purified samples are collected, concentrated and frozen in liquid nitrogen for later use.

Supplementary Note 3: Labeling of biomolecules

The protein is labelled based on cysteine maleimide chemistry. The protein is reduced with 1 mM Tris (2-carboxyethyl) phosphine (TCEP) pH 7.0 for 30 minutes at room temperature. Then the protein is equilibrated with ice cold phosphate saline buffer pH 6.7 by washing in a centrifugal 50kDa cut-off spin filter (3 times) which removes excess TCEP. Then 1-2 fold (mol) of dye (LD555 and LD655) is added. First the acceptor dye (LD655) is added and incubated for 2 minutes, then the donor dye (LD555) is added. The mix is incubated at room temperature for 2 h. The labelled protein is then purified with PD MiniTrap G25 columns (Cytiva). The column is washed thrice with Tyrode's buffer. Then the protein is added and the column centrifuged for 2 min, at 1000 g. The flow through was collected and used for the experiments.
To create heterodimers with one doubly labeled monomer and one unlabeled monomer, a monomer exchange was performed. The labeled protein is mixed with an unlabeled variant with a C-terminal zipper (Hsp90z_wt). A ratio of 1:20 (labeled:unlabeled) is used and final concentrations of 2 μM and 80 μM in Tyrode's buffer are used. The proteins are incubated at 43°C for 36 min with shaking at 300rpm in a thermocycler (Eppendorf). The heating destabilizes the C-terminal zipper and enables exchange of the monomers. Possible aggregates are removed subsequently by centrifugation at 14,000 g and 4 °C for 30 min.

Supplementary Note 4: Microinjection

Cell viability was tested by using pHrodo Red and Green AM intracellular pH indicator dyes (ThermoFisher Scientific). The cells used for injection were first calibrated for the dye response with different pH buffer conditions using the Intracellular pH calibration Buffer kit (ThermoFisher Scientific). Imaging of the cells was done on an Olympus microscope fitted with a HE90 dichroic cube. Images of the cell area of the different dishes were taken before dye addition. After addition of dye, washing and incubation with pH calibration buffers for the respective cell dishes an image of the same area was recorded. For cell viability test new cell dishes were taken and areas for injection were imaged in OptiMEM before injection. The following injection of cells were made with H1 buffer, then the cells were washed with preheated PBS (twice) and imaged in optiMEM. Images of the cell area of the different dishes was taken before dye addition. After addition of dye, washing and incubation with OptiMEM the respective areas of dishes were imaged. Uninjected area cells were also imaged as a control. For intensity measurements, mean of 10 cells were taken for each of the three time points (1st hour, 2nd hour, 3rd hour) for the 4 different pH measurements (pH 4.5, pH 5.5, pH 6.5, pH 7.5). For before and after measurements care was taken to measure the same cells and same region. All measurements were background corrected.

**Parameter**	**Value**
Speed (Coarse-mode) (µm/sec)	4000
Step injection speed (µm/sec)	50
Step injection distance (µm/sec)	20
Position speed (µm/sec)	1500
Injection axial	ON
Piezoxp. Speed (µm/sec)	300
Piezoxp. Distance (µm)	20
Piezoxp. axial	OFF
Angle of injection	45°

Table 3: Injector Parameters

Fixed cell experiments: The purified DNA/protein sample of 1nM is prepared in a vial with degassed buffer which is double filtered. Injection needle is filled with 5 µl of 1nM sample and fitted to the injector nozzle. Before injection, the cell area to be injected is imaged in the DIC, Green (555nm), Red(630nm) emission channels of the fluorescence microscope to check for any leakage or fluorescence abnormalities. The grid number on the ibidi dish is noted. The cells are then washed twice with DPBS 1ml (pre-warmed at 37°C) and then 1 ml of Opti-MEM is added, then cells within the marked grid area are injected. After injections, cell area within the grid is imaged again with DIC, green and red channels to confirm no leakage. Then the Opti-MEM is removed, cells washed with DBPS twice followed by addition of 500 µl of 4 percent Paraformaldehyde

(PFA). The dish is incubated for 20 mins in dark at room temperature. Then the PFA is washed twice with in DPBS and 2ml of Opti-MEM was added. The cells were then ready to be imaged.

Supplementary Note 5: HILO microscopy

For all *in cellula* measurements a home-built HILO microscope as depicted in Suppl.Fig. 2 was used. A theoretical depth of view calculation was done to estimate the minimum frame requirement for a molecule to be visible with a 70 ms exposure time and a certain diffusion constant. For a 100x Nikon objective with a 1.49 NA, the theoretical depth of field is 190 nm. The calculated depth of field d_{tot} as per the below parameters was found to be:

$$d_{\text{tot}} = \frac{\lambda n}{\text{NA}^2} + \frac{n \cdot e}{M \cdot \text{NA}} \tag{1}$$

Substituting given values:

$$d_{\text{tot}} = \frac{553\,\text{nm} \cdot 1.515}{1.49^2} + \frac{1.515 \cdot 14\,\mu\text{m}}{200 \cdot 1.49} = 449\,\text{nm} \tag{2}$$

Here, the refractive index $n = 1.515$; wavelength $\lambda = 553\,\text{nm}$; the variable $e = 14\,\mu\text{m}$ is the smallest distance that can be resolved by a detector placed in the image plane of the microscope objective, whose lateral magnification is $M = 200$. In the following we estimate the displacement of a single molecule (in z-direction) that can still be tracked, x_{tr}, assuming the molecule is first detected in the center of the focus. We start from the depth of field of the microscope as:

$$x_{\text{tr}} \approx \frac{1}{2} d_{\text{tot}} \tag{3}$$

Substituting (2) in (3), $x_{\text{tr}} \approx 224\,\text{nm}$ is the maximum possible mean squared displacement, where the molecule can be tracked, i.e., beyond this distance the particle might leave the focus and cannot be tracked. The mean squared displacement in one dimension, x^2, of a molecule is related to the diffusion coefficient D for a time t as:

$$t \approx \frac{x^2}{2D} \tag{4}$$

$$x \approx \sqrt{2Dt} \tag{5}$$

We can now calculate the mean squared displacement for a given diffusion constant and time. E.g. for $D = 0.05\mu\text{m}^2/\text{s}$ and t=10s we would obtain $x = 33$ nm. Therefore, most molecules would not leave the focus for such an experiment. **Correction factors** were estimated for esDNA and Hsp90_409C_601C. Both were labelled with LD655 and LD555 and both were biotinylated. The esDNA and Hsp90 were separately immobilized on PEG surfaces coated with Biotin-NHS PEG. Then the molecules were attached to the surface via biotin-Neutravidin binding and measured in H1 buffer pH 7.5 with Tris (5 mM Tris, 20 mM $MgCl_2$, 5 mM NaCl, pH 7.5) at 10pM for esDNA. The measurements were carried out with ALEX. The movies were analyzed using homewritten procedures based on IgorPro (wavemetrics), which is detailed in [3]. The obtained correction factors are given in Supplementary Table 6.

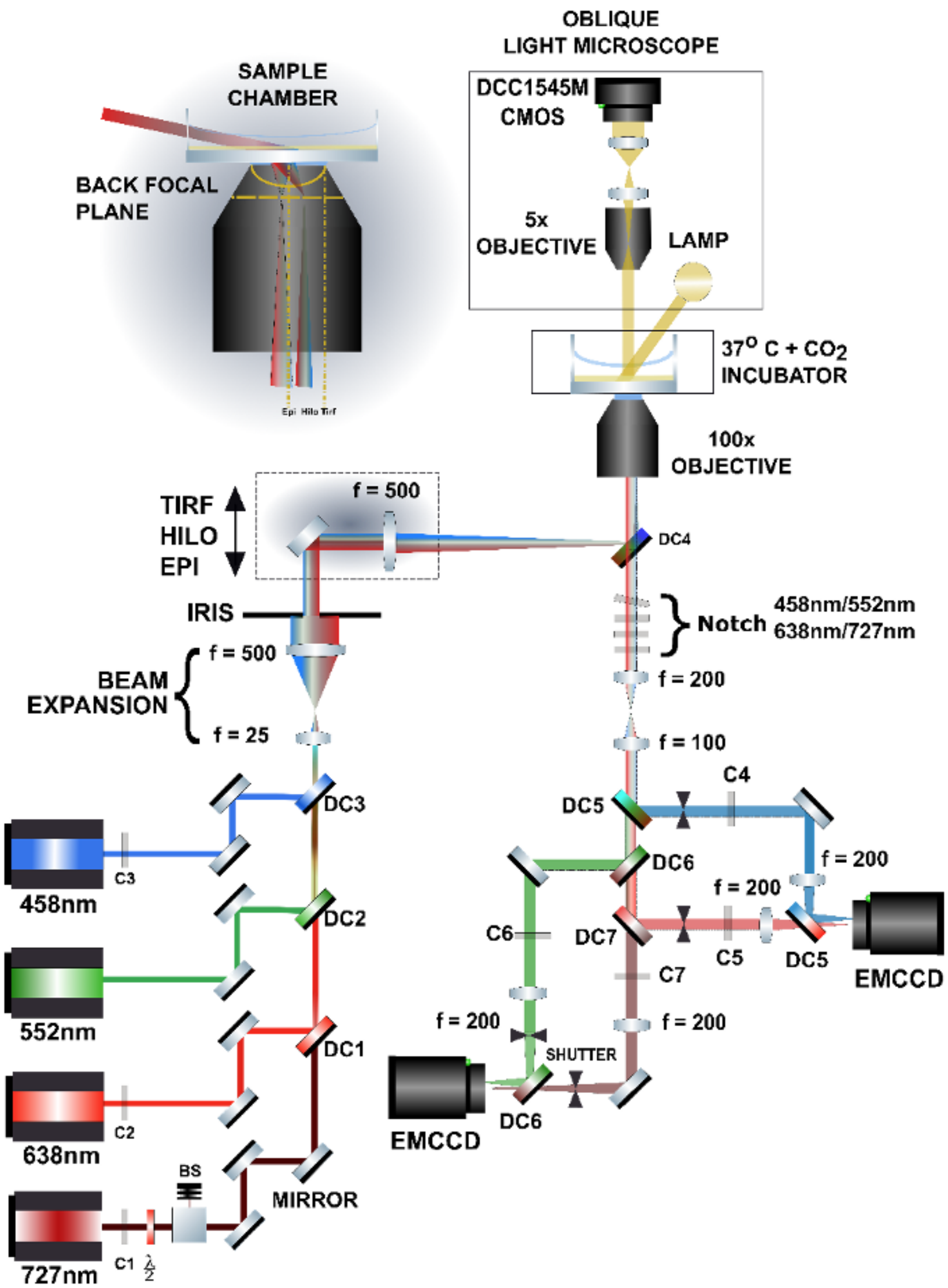
OBLIQUE
LIGHT MICROSCOPE
DCC1545M
CMOS
5x
OBJECTIVE
LAMP
SAMPLE
CHAMBER
BACK FOCAL
PLANE
Epi Hilo Tirf
37° C + CO_2
INCUBATOR
100x
OBJECTIVE
TIRF
HILO
EPI
f = 500
DC4
IRIS
Notch
458nm/552nm
638nm/727nm
f = 500
BEAM
EXPANSION
f = 25
f = 200
f = 100
C4
DC5
DC3
C3
458nm
DC6
f = 200
f = 200
DC2
C6
DC7
C5
DC5
EMCCD
552nm
C7
f = 200
f = 200
DC1
SHUTTER
C2
638nm
EMCCD
DC6
BS
MIRROR
727nm
C1
$\frac{\lambda}{2}$

Figure 2: Schematic representation of the 4-color HILO microscope equipped with an oblique light microscope for viewing cell injection area. All the optical and live-cell components are mounted on a vibration-damping optical table (RS2000, Newport). The following four lasers are used in our custom-built setup: 458nm (Obis 458LX, Coherent), 638nm (Obis 637LX, Coherent), 552nm (Obis 552LS, Coherent), 727nm (Obis 730LX, Coherent). The lasers are cleaned up using clearance filters and then overlaid using specific dichroic mirrors DC1, DC2 and DC3. An iris is positioned to select homogeneous illumination for all overlaid lasers. Subsequently the beam is expanded 20x by a Keplerian telescope and further a 2-inch iris cuts out the central illumination region. Further this beam is focused on to the back focal plane of the objective via a multiband Quad Line beam splitter DC4 using a 2-inch cylindrical lens mounted on a translation stage. This translation stage helps to shift the light path away from the optical axis (epi) to HILO and TIRF regions. The objective used is (CFI Apo TIRF 100x, Nikon) with NA=1.49. A set of notch filters are setup after the Quad Line beam splitter to block the excitation beam. The emission fluorescence is then directed to the detection box where the beam passes through a Keplerian lens with an adjustable optical slit (Owis GmbH). This helps to remove any off-axis beams and increases the magnification by a factor 2. Laser wavelengths are separated by dichroic mirrors DC5, DC6 and DC7. Corresponding emission filters C4, C5, C6, and C7 are placed in the emission pathway to cut off any residual excitation light. Additionally, a SH05 Ø1/2" Beam Shutter (Thorlabs) is placed after each emission filter to prevent bleed-through. The wavelengths 485nm and 637nm are separated from 552nm and 738nm and then focused separately on two EMCCD cameras (Xion Ultra 897 EX2 dua AR coated with fringe suppression, Andor Technology Ltd.) to facilitate multiple FRET-pair imaging. Finally, each beam is focused on to the full chip of the EMCCD detectors with aspheric lenses (Qioptiq) with 50.8 mm diameter. Sample chambers designed to integrate temperature, CO2 and humidity control (Okolab, Naples, Italy) are mounted on the microscope stage. For live-cell smFRET imaging, cells were grown in ibidi μ-dish 500 grid glass-bottom plates (ibidi GmbH, Munich). Filters: C1 - FL457.9/10 (ThorLabs), C2 - ZET635/10, C3 - 720/24 BrightLine HC, C4 - 525/50 BrightLine HC, C5 - 675/50 ET, C6 - 593/46 BrightLine HC, C7 - 810/90 ET Bandpass (rest from AHF Analysentechnik). Dichroics: DC1 - BS zt 670 rdc-xxrxt, DC2 - BS zt 561 RDC xrxt, DC3 - BS zt 473 RDC, DC4 - zt488/543/635/730rpc flat, DC5 - T 556 LPXR, DC6 - HC BS 649, DC7 - HC BS 740 (all from AHF Analysentechnik). Notch filters: 458 nm - ZET457NF,638 nm - ZET635NF, 727nm - ZET730NF (all from AHF Analysentechnik).

Supplementary Note 6: Data Analysis

The data analysis is done with a python based code called fret-analysis, previously described in [4] and available on GitHub. Analysis starts by running the Jupyter Notebook titled "01. Tracking". First, the microscope movies (TIFF file stacks) must be provided along with calibration files for the laser profile and channel alignment. Channel alignment is done prior to tracking. For this, threshold parameters for feature radius, mass and signal intensity need to be set so that fiducial markers (e.g. fluorescent beads) in the registration calibration file are recognized. The same procedure is repeated for the actual data files. Parameters should be chosen to locate single-molecule signals while minimizing the recognition of unspecific background fluorescence. The parameters used in this study are listed in table 4. For tracking the Crocker-Grier algorithm was chosen. Several tracking parameters can be adjusted (see table 5). The tracking results are saved into six separate files. Next, the notebook "02. Analysis AE" is executed. This notebook is based on the notebook "02. Analysis" provided in [4]. However, the built-in data correction is skipped (set $\alpha = 0, \delta = 0, \beta = 1, \gamma = 1$), because instead we employ our home written Igor Pro Software for correction. The updated data is again saved into six files. A manual trace selection is then carried out using the GUI tool "inspector_gui", which can be launched from the command line (python -m smfret_analysis.inspector_gui). This tool allows for visual inspection of each recognized and tracked particle. An overview of the following information is shown for each particle: its position in the three separate movies (donor, FRET and acceptor); intensity traces over time for each channel; and the SE plot. Based on this information, users can manually accept or reject traces for further analysis. Selections are saved by clicking the appropriate button in the interface. In the third step, the selected traces are exported as text files. This is done by running the adjusted notebook "03. Plots" up to the plot_summary function with the export="True" argument. These .txt files are subsequently used for further analysis in Igor Pro. To import the traces into Igor Pro, the function "Importer_ascii()" is used. To access the data the "SetNotes()" function must be called. The traces can then be viewed via the "show saved traces" panel. Each trace is then manually segmented into three regions: 1. Potential FRET – both dyes are present. 2. Single dye – one dye has bleached. 3. Background – both dyes have bleached. From this segmentation, SE 2D histograms and the corresponding 1D histograms can be generated. Correction can be done interactively. In this study, the correction factors were determined with in vitro measurements of esDNA, see Table 6.

Table 4: Localization and Tracking Parameters.

**Parameter**	**Donor**	**Acceptor**
**Registration options**		
Algorithm	Crocker-Grier	Crocker-Grier
Radius	2	2
Signal Threshold	1000	500
Mass Threshold	1000	1000
**Localization options**		
Algorithm	Crocker-Grier	Crocker-Grier
Radius	2	2
Signal Threshold	1000	500
Mass Threshold	20000	13000

Table 5: Linking of Particles Parameters.

**Parameter**	**Value**
Feature radius (feat_radius)	2
Link memory (link_mem)	20
Minimum length (min_length)	20
Background frame (bg_frame)	3
Background estimator (bg_estimator)	mean
Lag time	infinity

Table 6: Analysis Correction Factors.

**Correction factors**	**HILO**	**Confocal**
Leakage α	0.17	0.23
Direct Excitation δ	0.12	0.05
Excitation Efficiency β	1	0.7
Detection Efficiency γ	0.8	1

Supplementary Note 7: Example data and S vs E plots

In Suppl. Fig. 3 we show full distributions (S vs E plots and projections) for the DNA samples in vitor, in fixed cells and in live cells. Suppl. Fig. 4 shows additional example traces for DNA in living cells and suppl. Fig. 5 for Hsp90 in living cells.

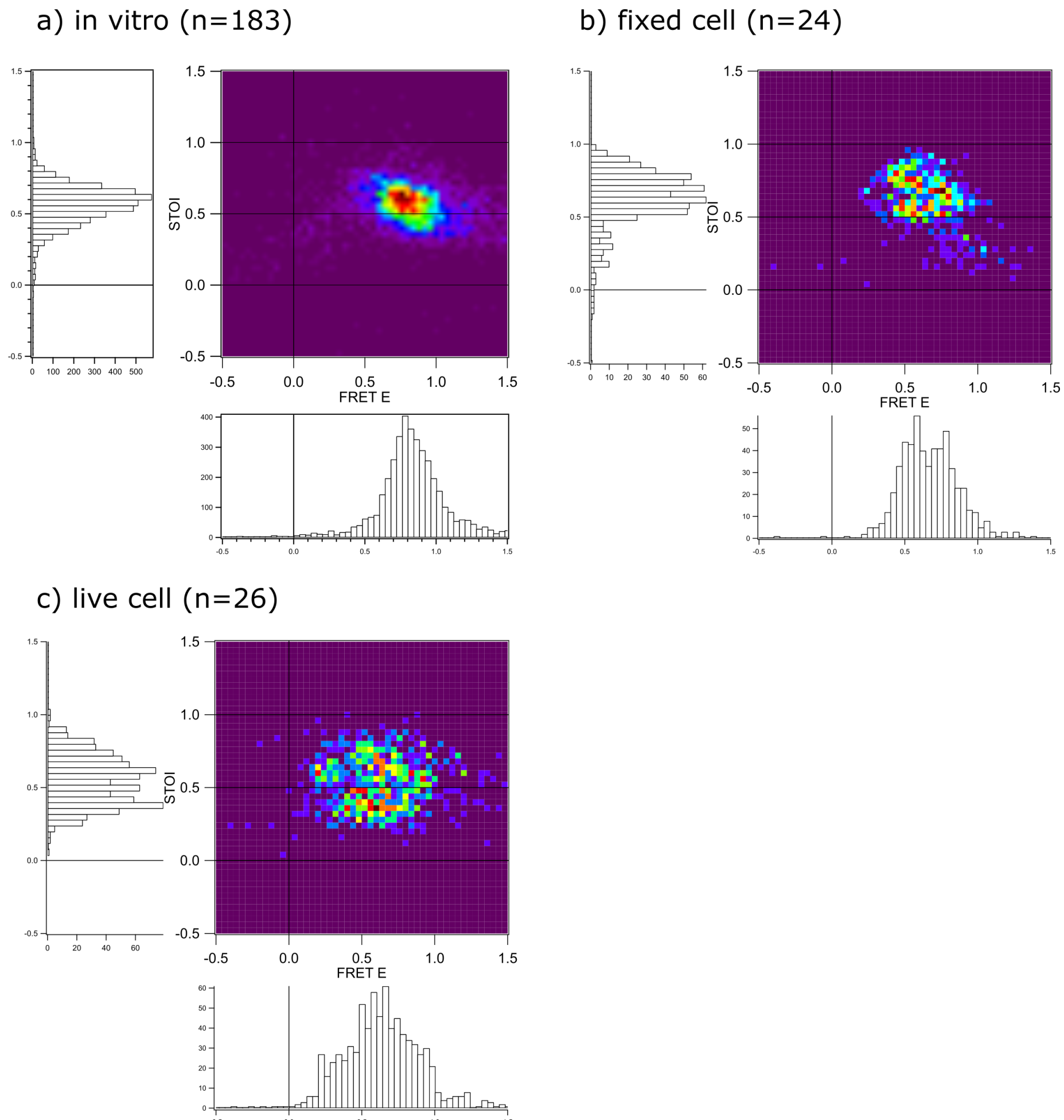

Figure 3: Combined distribution of esDNA smFRET data. a) In vitro data from a total of 183 traces obtained from 14 movies; b) Fixed cell data from a total of 24 traces obtained from 9 cells; c) Live cell data from a total of 51 cells. 1D histograms of stoichiometry (each left) and FRET efficiency (each bottom) and 2D histograms of stoichiometry vs FRET efficiency (each middle panel). Correction factors were applied.

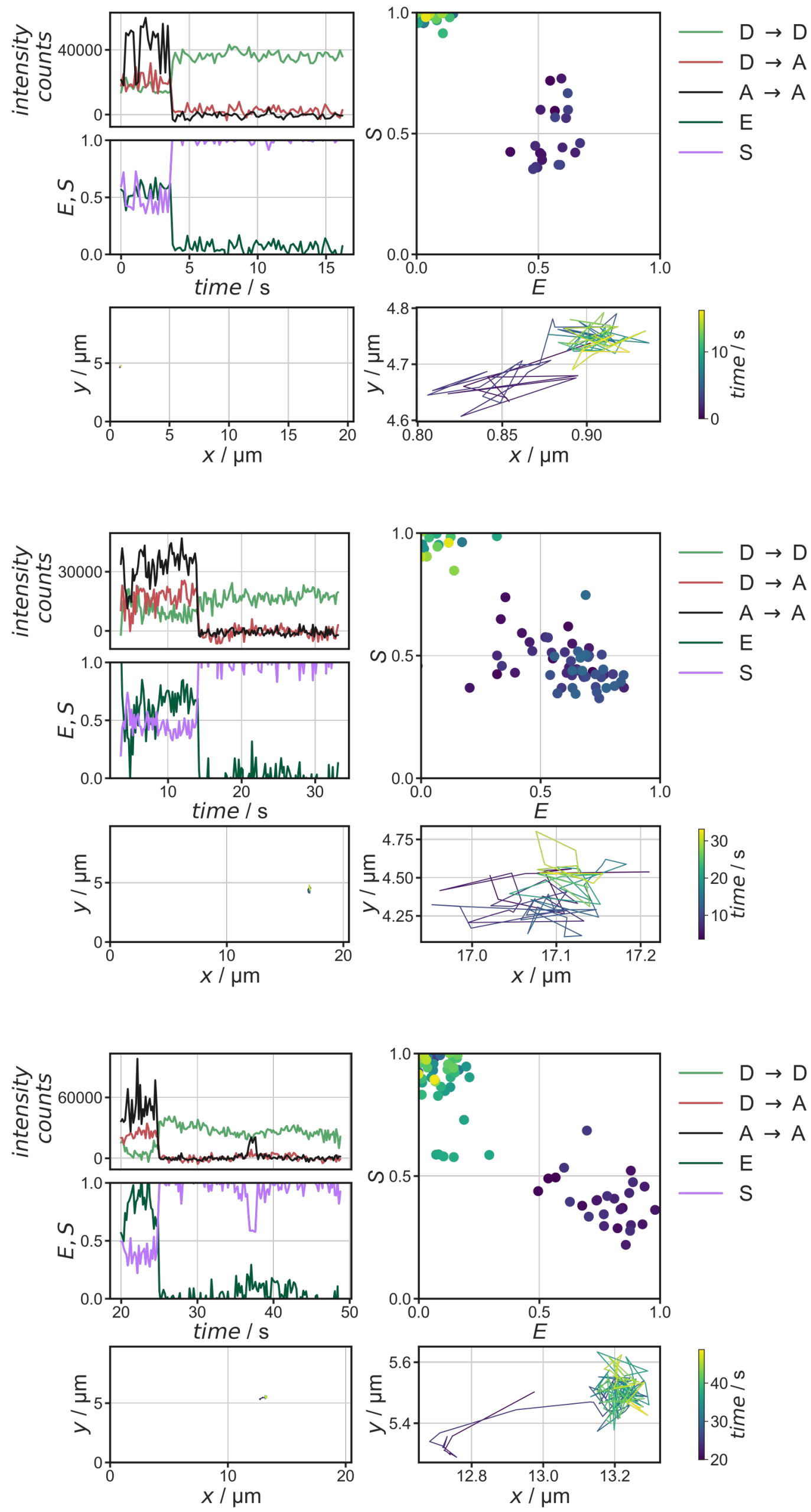

Figure 4: Example traces of live-cell smFRET measurements with esDNA. Intensity in donor, FRET and acceptor as well as FRET efficiency and stoichiometry over time. Corresponding E vs S plot and tracks. Data shown is raw data, i.e. no correction factors were applied. The top figure likely shows blinking of a second acceptor during the first four seconds, which demonstrates how important the ALEX is.

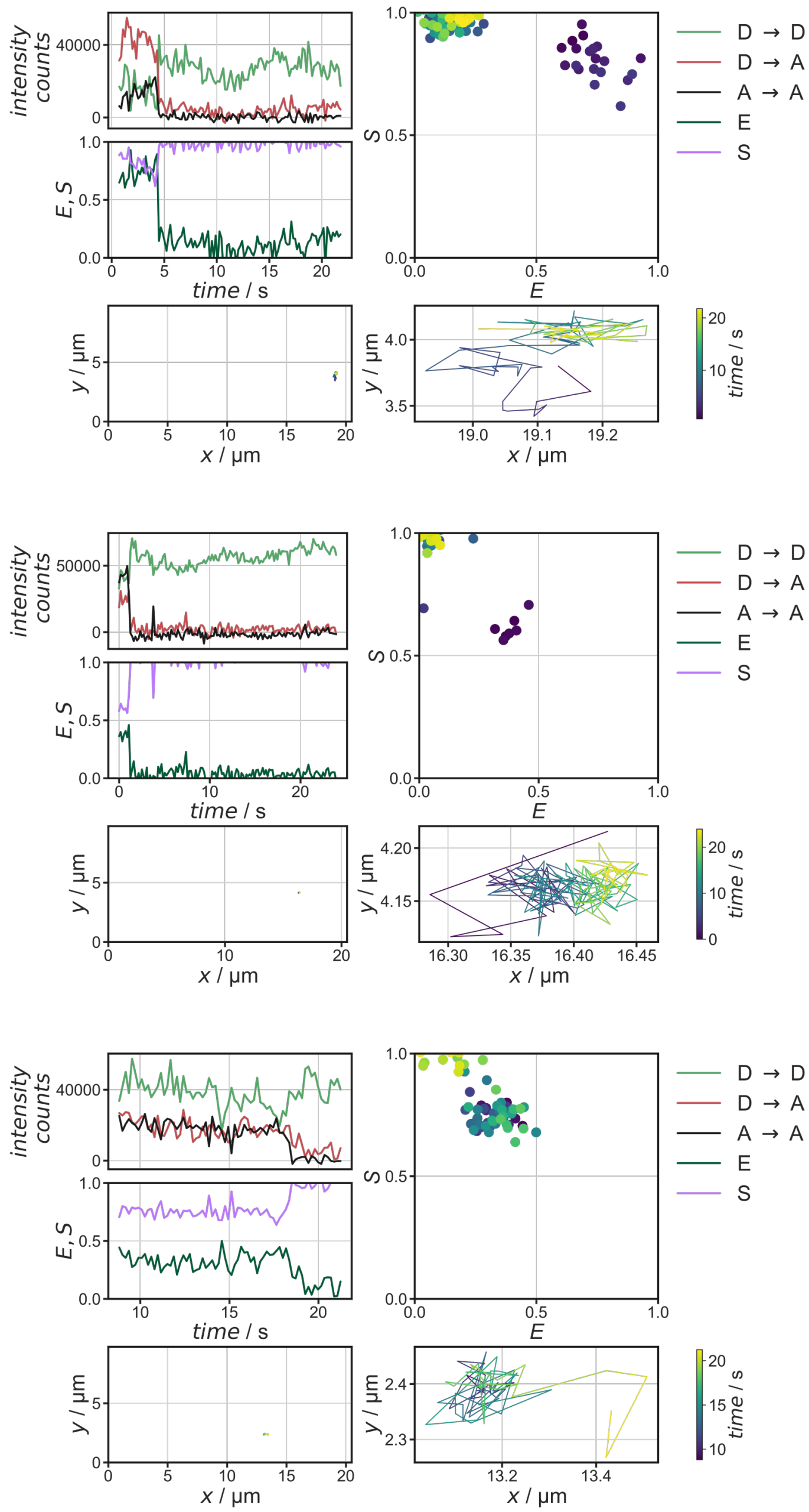

Figure 5: Example traces of live-cell smFRET measurements with Hsp90_409_601. Intensity in donor, FRET and acceptor as well as FRET efficiency and stoichiometry over time. Corresponding E vs S plot and tracks. Data shown is raw data, i.e. no correction factors were applied. This might in part explain the higher stoichiometry as expected (S around 0.5 is expected). Especially the beta factor (which affects S significantly) is difficult to determine.
